# Thermally Activated Negative Differential Resistance VO_*x*_ Memristor with Switchable Rate and Leaky Integrate-and-Fire Spiking Dynamics

**DOI:** 10.1021/acsnano.5c11481

**Published:** 2025-10-13

**Authors:** Li-Chung Shih, Zih-Siao Liao, Gennady Cherkashinin, Eszter Piros, Lambert Alff, Jen-Sue Chen

**Affiliations:** † Department of Materials Science and Engineering, 34912 National Cheng Kung University, Tainan 70101, Taiwan; ‡ Advanced Thin Film Technology Division, Institute of Materials Science, 26536 Technische Universität Darmstadt, Peter-Grünberg-Str. 2, Darmstadt 64287, Germany; § Program on Semiconductor Packaging and Testing, Academy of Innovative Semiconductor and Sustainable Manufacturing, 34912 National Cheng Kung University, Tainan 70101, Taiwan

**Keywords:** VO_*x*_ threshold switching neuron memristor, insulator-to-metal transition, negative differential resistance, spiking encoder, leaky integrate-and-fire neuron model

## Abstract

Spiking neural networks (SNNs) require neuron devices that are both compact and capable of supporting continuous-time and event-based dynamics. Here, we demonstrate a VO_*x*_-based threshold switching memristor (TSM) that intrinsically enables dual-mode operation, functioning as both a spiking encoder and a leaky integrate-and-fire (LIF) neuron. While such dual behavior is theoretically possible in volatile memristors, it has only been experimentally demonstrated in limited cases. It is achieved intrinsically in a single VO_*x*_-based device, arising from a thermally driven insulator-to-metal transition (IMT) within the VO_*x*_ layer, confirmed by temperature-dependent XRD and correlated with snap-back negative differential resistance (NDR) observed in electrical measurements. Integrated into a passive circuit, the device generates high-frequency spike trains under analog input and tunable LIF responses under pulsed stimulation. The device achieves a maximum spiking frequency of 570 kHz, a time-to-first-spike (TTFS) of 1.6 μs, and energy consumption as low as 4.7 nJ per spike. These results highlight the strong coupling between structural phase dynamics and neuronal functions, positioning the VO_*x*_ TSM as a promising platform for scalable neuromorphic hardware with biologically realistic spiking capabilities.

## Graphical abstract

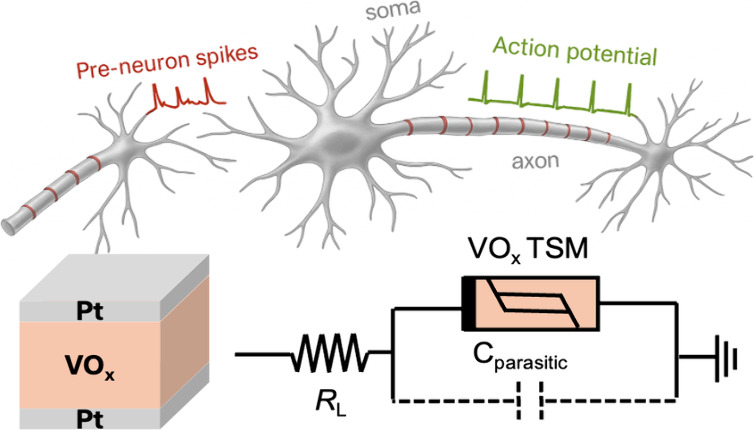

## 1 Introduction

Spiking Neural Networks (SNNs), regarded as the third generation of neural network models, have attracted increasing interest due to their ability to process information in a spatiotemporal and event-driven manner that closely resembles biological neural systems.^1,2^ Unlike conventional Artificial Neural Networks (ANNs), which rely on static inputs and synchronous, frame-based computation, SNNs transmit information through discrete voltage spikes.^3^ This spike-based encoding enables efficient processing of temporally distributed inputs, making SNNs particularly well-suited for event-driven and sparsely activated architectures. Their intrinsic ability to integrate multimodal signals while maintaining ultralow power consumption further highlights their suitability for deployment in energy-constrained applications such as wearable biosensors, real-time speech processing, and neuromorphic vision systems.^4,5^

Translating these functionalities into hardware implementations has become a key focus in neuromorphic research. This requires the development of compact, energy-efficient devices that can accurately reproduce key functions, including rate encoding and threshold-triggered firing.^6–10^ A widely adopted model that embodies the latter is the leaky integrate-and-fire (LIF) neuron, which captures event-driven computation through temporal integration and thresholding.^11–13^ While CMOS-based circuits offer mature design flexibility, as demonstrated by Wu et al.’s implementation of configurable neuronal functions using a “thyristor + resistor” circuit architecture,^14^ their high component density and substantial energy consumption remain significant challenges for large-scale neuromorphic integration. Alternatively, recent progress in volatile memristive devices, including those based on filamentary mechanisms such as Ag^15–17^ and Cu^18,19^ conductive bridges and ovonic threshold switching memristor,^20–22^ has demonstrated potential for emulating spiking behavior with simplified architectures. However, these approaches often suffer from stochastic switching dynamics, limited endurance, and high programming energy, which present challenges for reliable integration in neuromorphic systems. In contrast, Mott insulator-based threshold switching memristors, such as those utilizing VO_*x*_ ^23–25^ and NbO_*x*,_ ^26^ have received increasing attention due to their nonlinear thermal response, abrupt and intrinsic threshold switching characteristics, and low-voltage operation. Although volatile memristors with self-oscillatory dynamics theoretically offer a pathway to unify frequency encoding and LIF functionality within a single device, most experimental demonstrations to date have shown these features separately rather than concurrently.

In this work, we present a VO_*x*_ threshold switching memristor (TSM) as a compact and energy-efficient neuron device for hardware implementation of spiking neural networks (SNNs). The device’s electrical switching is driven by an insulator-to-metal transition (IMT) within the VO_*x*_ layer. This transition is experimentally confirmed through temperature-dependent X-ray diffraction (XRD) and current–voltage (*I*–*V*) measurements, revealing a clear correlation between the structural phase change and the corresponding electrical response. In alignment with these results, current-controlled *V*–*I* sweeps exhibit a continuous S-type response attributed to Poole–Frenkel conduction induced local phase transition and a sharp snap-back transition associated with the onset of IMT. These experimentally observed behaviors further support the role of IMT in enabling the nonlinear switching dynamics for dual-mode spiking emulation.

Leveraging these intrinsic switching properties, the VO_*x*_ TSM circuit successfully reproduces both spike encoding and LIF neuronal dynamics. As illustrated in Figure 1a, the membrane potential of a biological neuron accumulates during external stimulation and gradually decays in the absence of input. When the membrane potential reaches a critical threshold, it triggers an action potential, a phenomenon commonly referred to as neuronal firing.^27^ In order to mimic a biological spike input signal, a series of rectangular voltage pulses (*V*_in_) is applied as the input stimulus, enabling evaluation of the device’s integration capability.^28,29^ In the VO_*x*_-based circuit illustrated in Figure 1b, the spiking behavior arises from the charging and discharging of the parasitic capacitance (*C*_parasitic_) of VO_*x*_ TSM. As the *V*_in_ drives *C*_parasitic_ to accumulate charge, the voltage across the device (i.e., *V*_out_) gradually increases. Once *V*_out_ exceeds the threshold voltage (*V*_th_) of the VO_*x*_ TSM, the device switches to a conductive state and generates a sharp output current spike (*I*_out_) due to rapid capacitor discharge. Due to the definition of the measurement unit, these spikes are recognized as negative current values (details in the Section 4). This cyclic spiking response acts as a spiking encoder, as shown in Figure 1c. In contrast, under pulsed inputs, the circuit exhibits gradual integration of *V*_out_ during each pulse and decay between pulses due to leakage through the VO_*x*_ TSM. Once *V*_out_ reaches *V*_th_, the device fires and generates *I*_out_, effectively replicating the behavior of a LIF neuron, as demonstrated in Figure 1d.

**Figure 1:**
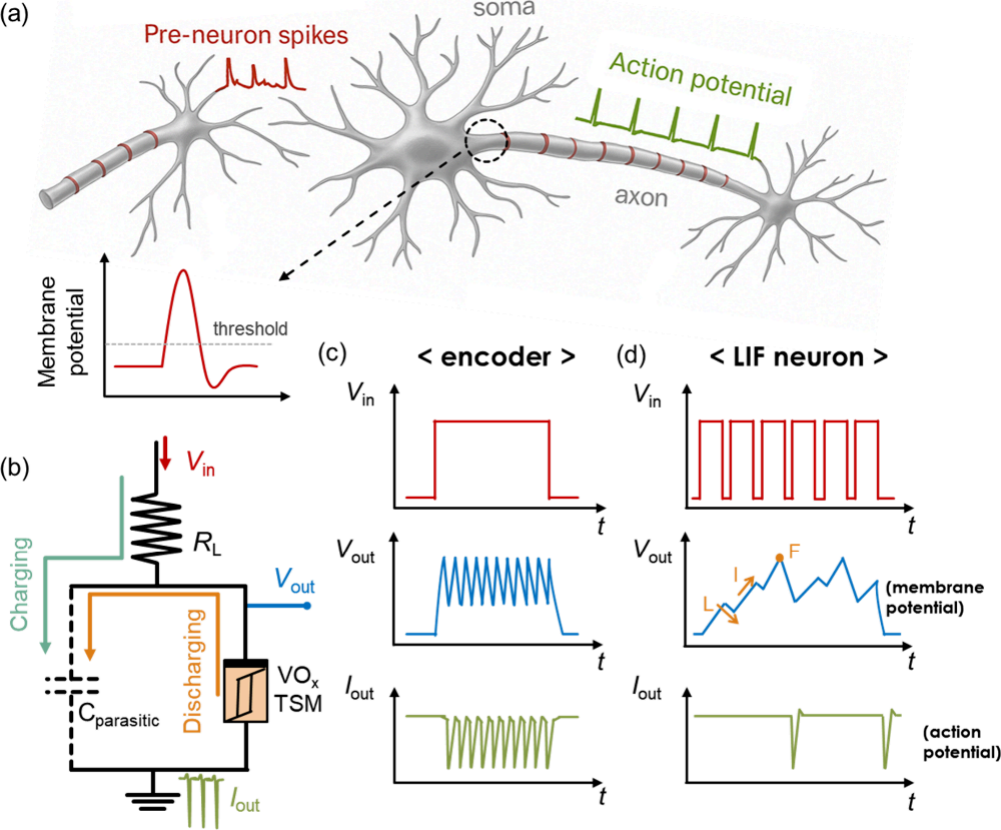
Implementation of spiking encoder and LIF neuronal models using a VO_*x*_-based TSM. (a) Schematic representation of a biological neuron. (b) Conceptual diagram of a VO_*x*_ TSM-based oscillatory neuron circuit. Representative input voltage (*V*_in_), output voltage (*V*_out_), and output current (*I*_out_) waveforms corresponding to (c) encoder and (d) LIF neuron applications.

## 2 Results and Discussion

### 2.1 VO_*x*_ Material Characterization

Figure 2a presents the schematic of the VO_*x*_ threshold switching memristor (TSM). Pt is selected at the top and bottom electrodes as it is believed that noble metals are chemically inert. This allows to avoid the formation of unwanted “parasitic” metal-oxide species at the oxide/electrode interface promoted by interfacial chemical reactions. In turn, such an improper surface impacts the conducting properties (i.e., the filament formation) between the top and bottom electrodes. The fabrication process flow for the Pt/VO_*x*_/Pt TSM device is illustrated in Figure S1 in the Supporting Information and detailed in the Section 4. The cross-sectional transmission electron microscopy (TEM) image of the fabricated device is shown in Figure 2b, revealing the columnar growth of the VO_*x*_ thin film with a measured thickness of 137 nm. High-resolution TEM (HRTEM) analysis, as indicated by label 1 in Figure 2b, reveals well-ordered lattice fringes with a *d*-spacing of 0.457 nm. This corresponds to the (200) plane of the VO_2_ phase, as shown in Figure 2c. To further confirm the crystal structure of the labeled region, a nanobeam electron diffraction (NBED) pattern along the [010] zone axis is presented in Figure 2d, confirming the crystalline monoclinic structure of VO_2_. In addition, the HRTEM image and NBED pattern along the [110] zone axis, corresponding to a differently oriented region of the VO_*x*_ film (label 2 in Figure 2b), are shown in Figure 2e,f, respectively. The observed *d*-spacings of 0.483 and 0.458 nm correspond to the (1–10) and (001) planes of the monoclinic VO_2_ phase. These results collectively verify the high crystalline quality of the sputter-deposited VO_*x*_ thin film.

**Figure 2:**
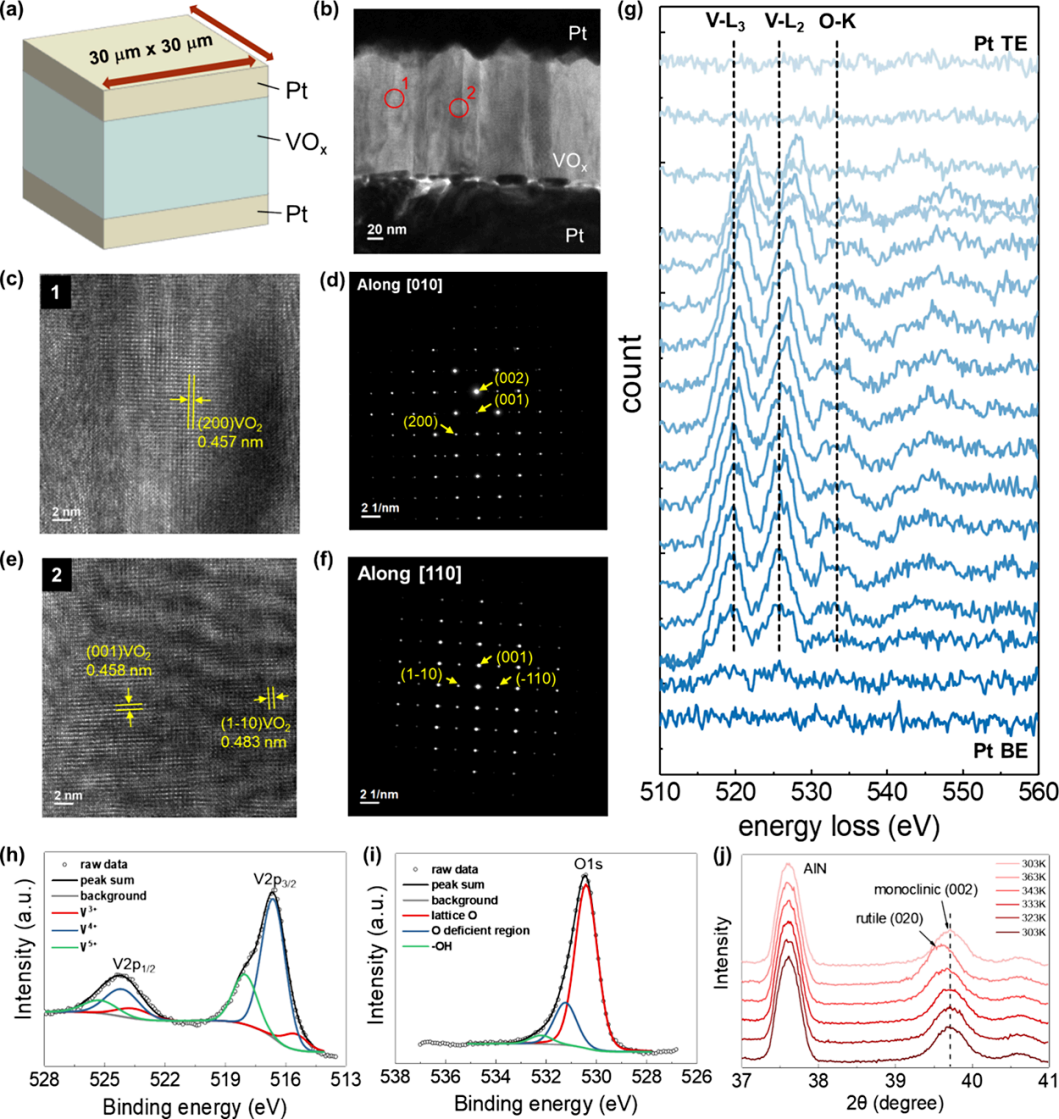
Material characterization of VO_*x*_ film. (a) Schematic illustration of Pt/VOx/Pt memristor with an electrode–oxide contact area of 30 × 30 μm^2^. (b) cross-sectional TEM image of Pt/VO_*x*_/Pt TSM device. (c, e) HRTEM images of the VO_*x*_ film and the corresponding NBED patterns acquired along (d) the [010] and (f) [110] zone axes at two different regions, respectively, confirming the crystalline nature of the VO_2_ monoclinic phase. (g) EELS line-scan spectra across the VO_*x*_ layer. (h) V 2p and (i) O 1s photoelectron spectra measured by in-situ XPS of the as-deposited VO_*x*_ film. (j) Temperature-dependent X-ray diffraction (XRD) patterns of the VO_*x*_ film on a Si substrate during heating (303–363 K) and subsequent cooling, demonstrating a reversible phase transition.

Elemental distribution analysis using high-angle annular dark-field scanning transmission electron microscopy (HAADF-STEM) and energy-dispersive X-ray spectroscopy (EDS) mapping is shown in Figure S2 (Supporting Information). The HAADF-STEM image demonstrates a sharp interface between the VO_*x*_ and Pt electrodes. The EDS elemental maps clearly show distinct spatial distributions of vanadium, oxygen, and platinum with well-defined boundaries. Furthermore, the EDS line-scan profiles across the Pt/VO_*x*_/Pt structure are shown in Figure S3 (Supporting Information), providing a more quantitative evaluation of elemental distribution along with the film thickness.

To characterize the structural quality and vanadium valence state of the VO_*x*_ film at the atomic scale, electron energy loss spectroscopy (EELS) and X-ray photoelectron spectroscopy (XPS) are performed. Figure 2g shows line-scan EELS spectra collected along the cross-section of the device, highlighting the spatial variation in vanadium oxidation states. Near the bottom Pt electrode, the V L_3_ and L_2_-edge peaks are located at approximately 519.4 and 525.6 eV, respectively.^30,31^ Notably, the corresponding L_3_-edge peak near the top electrode shifts by approximately 2.1 eV toward higher binding energy, reaching 521.6 eV. This shift indicates an increased oxidation state of vanadium in the upper region of the film. Additionally, the observed variation in peak positions is consistent with previously reported EELS spectra for vanadium oxides, specifically between VO_2_ and V_2_O_5_. These findings confirm that the dominant oxidation state in the VO_*x*_ film is V^4+^, with a higher valence state present near the top electrode. This gradient in oxidation state is attributed to the ex-situ deposition process of the Pt top electrode, which may introduce additional oxygen exposure during fabrication.

In-situ X-ray photoelectron spectroscopy (XPS) analysis, presented in Figure 2h,i and detailed in Supplementary Note 1 (in the Supporting Information), confirms that the as-deposited VO_*x*_ thin film exhibits a mixture of oxidation states. The V 2p_3/2_ photoelectron peak appears at binding energies of 515.3, 515.8, and 517.2 eV, which are associated with the V^3+^, V^4+^, and V^5+^ oxidation states, respectively.^32^ The O 1s photoelectron spectrum shows an asymmetry to higher binding energy, thereby evidencing more than one oxidation state contributes to the spectrum (Figure 2i). The best fitting results on the O 1s photoelectron spectrum were obtained for three components located at 530 eV (V–O bonding), 531.5 eV (oxygen-deficient regions), and a minor contribution at ∼533 eV likely associated with traces of −OH groups on the surface. The relative atomic percentages of the vanadium oxidation states are provided in Table S1 (Supporting Information), indicating that V^4+^ (VO_2_) is the dominant component. This finding aligns well with the NBED and EELS results, further confirming the prevalence of VO_2_ within the mixed-phase VO_*x*_ film. Based on these combined characterizations, the overall stoichiometry of the film is estimated as VO_2.2_.

Figure 2j illustrates 2θ-ω scans temperature-dependent structural phase transition of the VO_*x*_ on Si substrate with 2θ between 37 and 41° and at temperatures ranging from 303 to 363 K, covering the range over which the material lattice structure is changing. The AlN peak labeled in Figure 2j originates from the AlN substrate used in the temperature-dependent XRD setup. Although unrelated to the VO_*x*_ thin film, this peak provides a stable reference for calibrating the peak shifts of VO_*x*_ during heating and cooling. By aligning the AlN peaks, the structural phase transition of VO_2_ can be clearly observed through the temperature-dependent shift of the diffraction peak. The diffraction peak of the monoclinic VO_2_ (002) phase at a low-temperature range undergoes a change to the tetragonal rutile (020) phase at a high temperature (ICDD PDF card no. 00-033-1441 and 00-044-0253). Upon cooling to 303 K, a transition from the rutile to the monoclinic phase of VO_2_ is observed via the formation of V–V dimers along the *c* axis. This temperature-dependent XRD result reveals the reversible electron–lattice interaction (Peierls transition) of our VO_*x*_ layer.^33^

In addition, Figure S4 (in the Supporting Information) shows the temperature-dependent resistance curve of the VO_2_ TSM device during the heating and cooling process obtained at 0.1 V. The device exhibits insulation properties with a resistance of ∼7468 Ω at room temperature. The resistance change of the device is greater than 2 orders of magnitude when undergoing the IMT within the temperature range of 330–343 K. The IMT temperatures for thermal processes are determined by the first derivative of resistance d­(log­(*R*))/d*T* versus temperature (the inset in Figure S4 in the Supporting Information), which are 339 K upon heating and 337 K upon cooling, respectively. Importantly, the IMT plays a critical role in enabling the fast and reversible switching characteristics observed in our VO_*x*_ TSM devices. These findings indicate that the device functionality is predominantly governed by the intrinsic physical properties of VO_2_.^34,35^ Note that the narrow thermal hysteresis loop observed in our devices originates from the nonstoichiometry nature of the VO_*x*_ film, as revealed by XPS analysis (Figure 2h). The incorporation of V^3+^ and V^5+^ effectively dopes the VO_2_ lattice, introducing lattice strain, which in turn suppresses the intrinsic hysteresis.^36^

### 2.2 Electrical Characteristics and Physical Mechanism of VO_*x*_ TSM

Figure 3a presents the *I*–*V* characteristics of the VO_*x*_ threshold switching memristor (TSM) under a compliance current (CC) of 1 mA across 100 cycles. Symmetric hysteresis loops are observed for both positive and negative bias polarities. The device switches from a high-resistance state (HRS) to a low-resistance state (LRS) once the applied voltage exceeds the threshold voltage (*V*_th_), approximately 1.2 and −1.0 V for positive and negative sweeps, respectively. It returns to the HRS when the voltage drops below the hold voltage (*V*_h_), around 0.6 and −0.55 V. This resistive switching behavior arises from the insulator-to-metal transition (IMT) in VO_2_, which is triggered by Joule heating and involves intertwined structural and electronic phase changes, as evidenced in Figures 2f and S4 (in the Supporting Information).^37–39^

**Figure 3:**
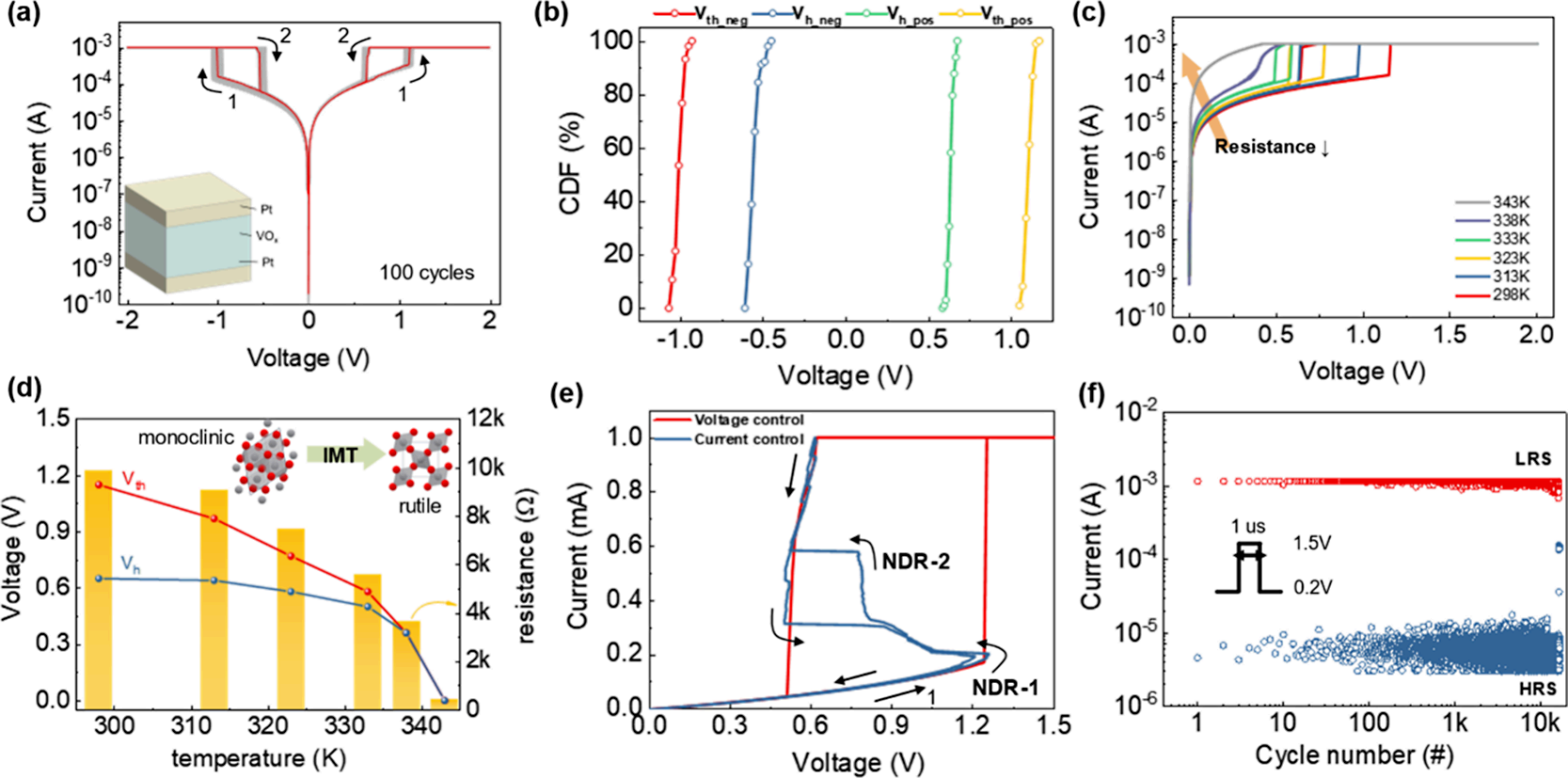
Electrical characteristics of VO_*x*_ TSM. (a) *I*–*V* characteristics of the VO_*x*_ TSM device repeated for 100 cycles. (b) Cumulative distributions of positive threshold voltage (*V*_th_pos_), positive holding voltage (*V*_h_pos_), negative threshold voltage (*V*_th_neg_), and negative holding voltage (*V*_h_neg_). (c) Temperature-dependent *I*–*V* characteristics of VO_*x*_ TSM and (d) corresponding extracted *V*_th_, *V*_h_, and resistance measured at 0.1 V as a function of temperature. (e) Comparison of current-controlled *V*–*I* (blue) and voltage-controlled *I*–*V* (red) responses at room temperature. NDR-1 and NDR-2 are two regions of negative differential resistance. (f) Endurance measurement of VO_*x*_ TSM under repeated 1.5 V pulsed operation (pulse width: 1 μs).

Figure 3b displays the cumulative distributions of positive and negative threshold/hold voltages, including *V*_th_pos_, *V*_h_pos_, *V*_th_neg_, and *V*_h_neg_ in 100 repeated cycles. The coefficient of variation (*C*_v_) defined by the ratio of the standard deviation (σ) to the mean value (μ) of *V*_th_pos_, *V*_h_pos_, *V*_th_neg_, and *V*_h_neg_ are 2.2, 11.5, 2.6, and 5.9%, respectively, showing low cycle-to-cycle (C2C) variations. Furthermore, Figures S5 and S6 (in the Supporting Information) summarize device-to-device (D2D) variability across 15 devices, each tested with 20 consecutive cycles. The D2D C_v_ values for *V*_th_pos_, *V*_h_pos_, *V*_th_neg_, and *V*_h_neg_ are 7.7, 12.5, 8.2, and 13.7%, respectively, as listed in Table S2 (in the Supporting Information). These results indicate that our devices show no noticeable degradation, demonstrating their reproducibility and stability for integration into physical networks.

To investigate the switching mechanism, Figure 3c shows the temperature-dependent *I*–*V* characteristics. As the temperature increases, the resistance of the VO_*x*_ TSM in the prethreshold switching regime decreases, accompanied by a reduction in both the *V*_th_ and *V*_h_, as illustrated in Figure 3d. This behavior suggests a thermally induced phase transition in the VO_*x*_ layer, from a monoclinic insulating phase to a rutile metallic phase. The reduction in switching voltages suggests a lowered energy barrier for the IMT at elevated temperatures. At approximately 343 K, the device transitions into a fully conductive state, maintaining a low-resistance path under applied bias, consistent with complete transformation into the thermodynamically stable rutile phase. Upon cooling, the device recovers its pristine switching characteristics, indicating that the phase transition is reversible and thermally driven, as shown in Figure S7 (in the Supporting Information). This electrical behavior aligns well with the temperature-dependent XRD results shown in Figure 2h, corroborating the strong link between structural phase evolution and electrical switching.

In addition, Figure 3e compares the current-controlled *V*–*I* (blue) and voltage-controlled *I*–*V* (red) characteristics of our VO_*x*_ TSM obtained at room temperature. Under current-controlled operation, the device exhibits two distinct regions of negative differential resistance (NDR): an S-type NDR (NDR-1) at low current levels and a snap-back NDR (NDR-2) at higher currents.^40–44^ This two-stage NDR can be attributed to the nonstoichiometric nature of our VO_*x*_ thin film. As revealed by XPS analysis in Figure 2h, the film contains not only V^4+^ but also other valence states such as V^3+^ and V^5+^. The incorporation of V^3+^ and V^5+^ ions induce lattice strain, resulting in a lower phase transition temperature in localized defect-rich VO_*x*_ regions.^36^

When the incremental current is supplied, the I–V trace is initially dominated by the Poole-Frankel (P–F) conduction mechanism. Its inherent positive thermal feedback causes rapid Joule heating, raising the local temperature to the threshold required to trigger a phase transition in localized defect-rich (V^3+^/V^5+^) VO_*x*_ regions, which manifests as the first NDR (NDR-1). The P–F conduction mechanism contributes to sustained Joule heating. As the device temperature increases, the thermal runaway effect induced by the P–F mechanism intensifies, eventually providing enough energy to trigger a phase transition in the main VO_2_ material. This is then responsible for the second, more significant drop in resistance, observed as NDR-2. During the reverse sweep, the device remains in the metallic state until the thermal energy dissipates and is no longer sufficient to maintain the rutile phase, triggering a transition back to the monoclinic insulating state. The voltage drop associated with this return transition in the current-controlled characteristic corresponds closely to *V*_h_ observed in the voltage-controlled characteristic.

Moreover, the switching speed of our VO_*x*_ TSM is evaluated using a 1.5 V voltage pulse, as shown in Figure S8 (in the Supporting Information). The device switches from the off-state to the on-state within approximately 70 ns, and from the on-state back to the off-state in about 20 ns, demonstrating high-speed switching behavior that is comparable to previously reported VO_*x*_-based devices.^45^ Additionally, endurance measurements under repeated pulsed operation at 1.5 V with a pulse width of 1 μs for over 1.5 × 10^4^ cycles, shown in Figure 3f. It is observed that both HRS and LRS begin to degrade after approximately 17,000 switching cycles. Beyond this point, incomplete recovery of HRS leads to a pronounced reduction in the on/off resistance ratio. This degradation is attributed to cumulative Joule heating during repeated pulsing at 1.5 V with 100 ns intervals, where the short interpulse relaxation limits heat dissipation and causes localized thermal accumulation.^46^ The resulting temperature rise promotes an IMT transition in the VO_*x*_ layer, driving a local transformation from the monoclinic insulating phase to the conductive rutile phase. This thermally driven evolution is consistent with Figure 3d, which shows a decrease in resistance with increasing temperature.

### 2.3 Implementation of VO_*x*_-Based Oscillation Encoder Circuit

The configuration of the oscillation circuit based on the VO_*x*_ threshold switching memristor (TSM) and its associated charging and discharging processes is illustrated in Figure 4a. The VO_*x*_ TSM is connected in series with a load resistor (*R*_L_), and the voltage drop (*V*_out_) across the TSM is monitored using an oscilloscope. The role of *R*_L_ and its relationship to device resistance is detailed in Supplementary Note 2 (in the Supporting Information). Upon applying an input voltage, the parasitic capacitance (*C*_p_) of the device, which functions as a parallel-connected capacitor, begins to charge. Once *V*_out_ across the capacitor exceeds the *V*_th_ of the VO_*x*_ TSM, the device switches to LRS. As a result, a current spike (*I*_out_) is generated, and the capacitor discharges through the conductive TSM. As the capacitor voltage subsequently drops below *V*_h_, the TSM returns to its HRS. This reversible switching behavior of the VO_*x*_ TSM enables the circuit to realize the spiking encoder by converting continuous input signals into spiking outputs. Figure 4b–d presents the circuit’s response without an external parallel capacitor under positive triangular input voltages ranging from 2 to 10 V and 5 to 10 V, for *R*_L_ values of 10, 15, and 22 kΩ, respectively. The corresponding circuit response under negative triangular input voltages for various *R*_L_ values is shown in Figure S9 (in the Supporting Information).

**Figure 4:**
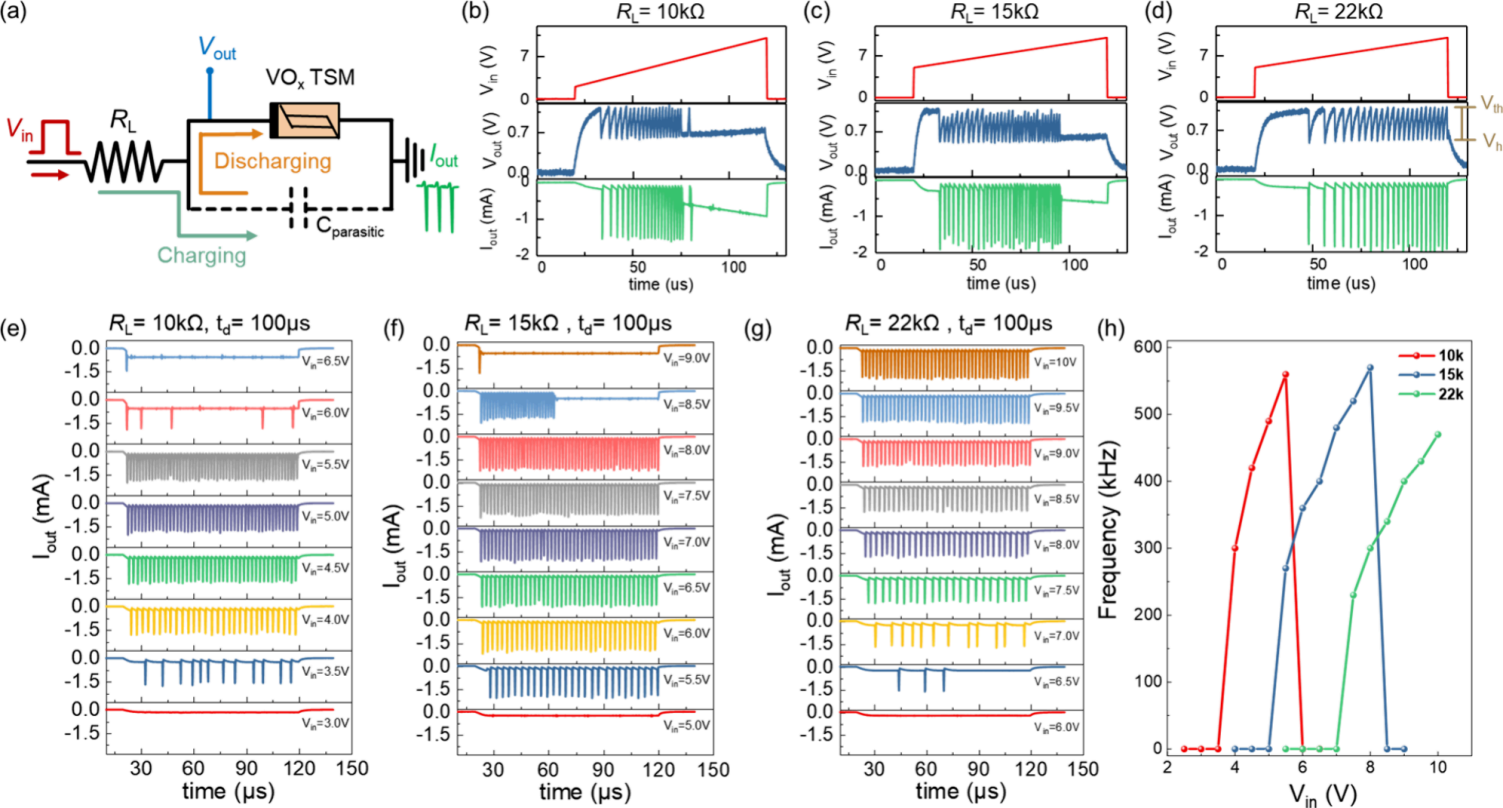
VO_*x*_-based implementation of the VO_*x*_ TSM encoder. (a) Schematic of the oscillation circuit utilizing a VO_*x*_ TSM. (b–d) Output oscillation responses of *V*_out_ and *I*_out_ under positive triangular *V*_in_ with varying *R*_L_. (e–g) Corresponding *I*_out_ responses under rectangular *V*_in_ with different *R*_L_ values. (h) Dependence of spiking frequency on *V*_in_ and *R*_L_. The spiking frequency increases with higher *V*_in_ and decreases with increasing *R*_L_. The data without stable spiking within 100 ms are assigned a frequency of 0 Hz.

To further evaluate the *I*_out_ response of the oscillation circuit, a pulsed input voltage (*V*_in_) with varying amplitude and a fixed pulse width (*t*_d_) of 100 μs is applied across different *R*_L_, as shown in Figure 4e–g. The resulting spiking frequencies of *I*_out_ and corresponding *V*_out_ waveforms are summarized in Figure 4h and S10 (in the Supporting Information), respectively. As *R*_L_ increases, the input current decreases, leading to a slower charging rate of *C*_parasitic_ of VO_*x*_ TSM and a reduction in spiking frequency. In contrast, higher *V*_in_ enhances the charging current, thereby accelerating capacitor charging and increasing the spiking rate.

A maximum spiking frequency of approximately 570 kHz is consistently achieved when *V*_in_ is 5.5 and 8 V for *R*_L_ of 10 and 15 kΩ, respectively. Although *R*_L_ and *V*_in_ differ, this convergence results from a balance between the charging and discharging dynamics of the circuit. Increasing *V*_in_ accelerates the charging rate of *C*_parasitic_ of VO_*x*_ TSM, thereby reducing the integration time required to reach the switching threshold. However, this is accompanied by a prolonged discharging phase. The observed frequency saturation arises when these opposing effects are balanced, defining an upper limit governed by the intrinsic switching and recovery characteristics of the VO_*x*_ TSM. It is important to note that when the input voltage is lower (or higher) than the spike-triggering threshold, the resulting insufficient (or excessive) charging current fails to switch the VO_*x*_ TSM to LRS (or HRS).

Moreover, in biological systems, signals are typically transmitted in continuous, analog form, and sensor outputs often reflect such characteristics. To replicate this, sinusoidal input voltages are applied to a VO_*x*_-based oscillation circuit configured with the *R*_L_ of 15 kΩ, as shown in Figure S11 (in the Supporting Information). The corresponding spiking frequency is displayed in the fourth panel, where the frequency-time curve also adopts a sinusoidal pattern. The stability and reliability of the VO_*x*_ TSM are further confirmed by the consistent spiking frequency observed under repeated sinusoidal inputs. The input-dependent spiking frequency indicates that the circuit can serve as a neural encoder, where analog inputs are represented as spike rates, emulating a biologically inspired rate coding scheme.^27,47–49^

### 2.4 Implementation of the LIF Model Using VO_*x*_-Based Neuron Circuit

The development of highly compact artificial neurons and synapses, especially those that capture the dynamic behavior of key biological elements, is of great significance for neuromorphic computing inspired by the structure and principles of the human brain.^8,12,50^ LIF artificial neurons, in particular, mimic neuronal functions associated with the accumulation and leakage of electric charge across the cell membrane. In biological neural networks, neurons receive input signals from other neurons through synaptic connections and generate action potential when the membrane potential reaches a threshold. Similarly, in artificial neurons, when the accumulated voltage across a capacitor reaches the threshold, the neuron fires and outputs spike pulses.^27,51,52^

Figure 5 illustrates the implementation of the LIF model using our VO_*x*_-based oscillation circuit, which leverages the *C*_parasitic_ inherent in the VO_*x*_ TSM device (Figure 5a). When multiple fast input voltage pulses are applied, the output voltage (*V*_out_) dynamically increases and decreases due to the charging and discharging of *C*_parasitic_ (Figure 5b). Once *V*_out_ reaches the threshold voltage (*V*_th_ ≈ 1.2 V) of the VO_*x*_ TSM, a rapid voltage drop is observed as the capacitor discharges through the now-conductive TSM. This dynamic closely mimics the behavior of a biological neuron, where the membrane potential integrates incoming stimuli over time and decays in their absence. When the accumulated potential surpasses a threshold, the neuron fires an action potential, followed by a rapid repolarization. In our system, the charge and discharge of *C*_parasitic_ emulate this membrane potential behavior, and the firing rate can be finely controlled by adjusting the input pulse amplitude, width (*t*_d_), and interval (Δ*t*).

**Figure 5:**
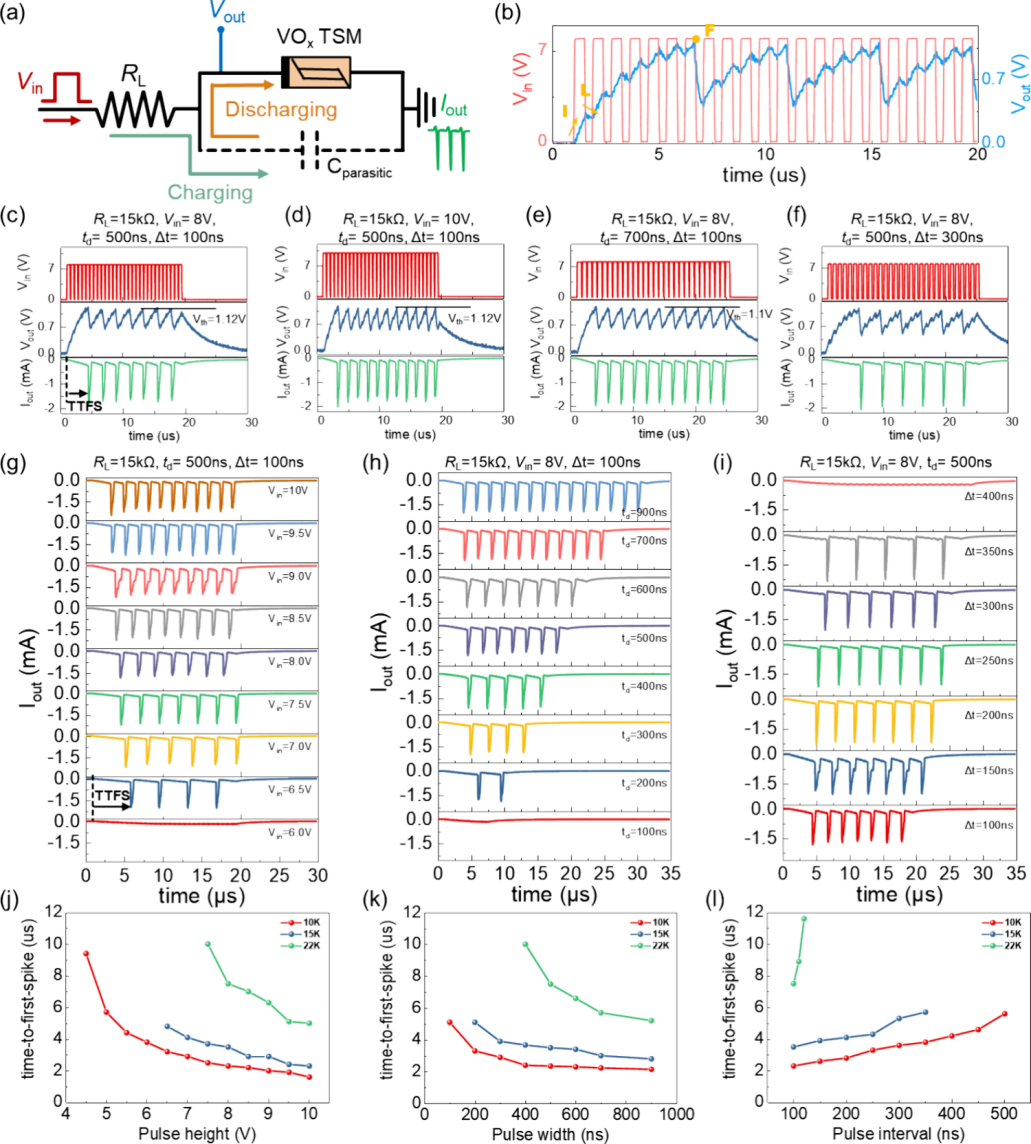
VO_*x*_-based implementation of the LIF neuron model. (a) Schematic of the LIF circuit utilizing a VO_*x*_ TSM. (b) Representative *V*_out_ response under pulsed *V*_in_, demonstrating characteristic leaky, integrative, and firing behavior. (c–f) Output responses of *V*_out_ and *I*_out_ under a train of 30 input pulses with a fixed *R*_L_ of 15 kΩ, showing the effects of varying *V*_in_, pulse width (*t*_d_), and pulse interval (Δ*t*). Corresponding *I*_out_ responses under variations in (g) *V*_in_, (h) *t*_d_, and (i) Δ*t* with *V*_in_ pulse number fixed at 30. Extracted TTFS as a function of (j) *V*_in_, (k) *t*_d_, and (l) Δ*t* for different *R*_L_ values, based on data in (g–i). TTFS increases with larger *R*_L_, indicating a slower integration process due to reduced input current.

Figure 5c–f show the input voltage (*V*_in_), the voltage across the TSM (*V*_out_), and the current response (*I*_out_) as functions of time, using the circuit shown in Figure 5a with a fixed *R*_L_ = 15 kΩ. These results clearly illustrate the dynamic switching process of the VO_*x*_ TSM device. The current output remains low and relatively constant between spikes, indicating the HRS of the VO_*x*_ TSM before threshold switching. Due to the voltage division effect (*R*_HRS_ > *R*_L_), most of the input voltage initially drops across the VO_*x*_ TSM, enabling *C*_parasitic_ to charge. During each *V*_in_ pulse, the capacitor incrementally stores charge, analogous to the voltage sweep in *I*–*V* measurements. Once *V*_out_ across the capacitor exceeds *V*_th_ of the VO_*x*_ TSM, sufficient local Joule heating is produced to trigger the IMT. As a result, the device abruptly switches to its LRS, producing a sharp current spike that corresponds to an artificial neuron firing event. It is worth noting that increasing either the amplitude or duration of *V*_in_ reduces the number of pulses required to accumulate sufficient charge on the parasitic capacitance, thereby shortening the time-to-first-spike (TTFS). Importantly, these variations in pulse parameters do not affect the intrinsic firing threshold voltage of the device, highlighting the stability and reliability of the VO_*x*_ TSM’s switching characteristics.

On the other hand, when the pulse interval is set to Δ*t* = 100 ns (Figure 5c–e), the leaky behavior is minimal, deviating from the typical characteristics of the LIF model. This behavior is consistent with the estimated RC time constant of approximately 112 ns, based on a parasitic capacitance of ∼14 pF (Figure S12 in the Supporting Information). Since Δ*t* is shorter than the RC time constant in this scenario, charge leakage is limited, and the system exhibits dominant integration behavior. Together, these experimental results confirm that the temporal firing behavior of the circuit can be precisely modulated through input pulse engineering, thereby validating the LIF functionality of the VO_*x*_-based system.

To further assess the performance of the VO_*x*_ TSM device in the LIF model, a series of pulse trains is applied to the VO_*x*_-based circuit. Each pulse train consisted of 30 pulses with varying parameters, including pulse amplitude (*V*_in_, 6–10 in 0.5 V increments), pulse width (*t*_d_, 100–900 in 100 ns increments), and pulse intervals (Δ*t*, 100–400 in 50 ns increments). Figure 5g–i shows the *I*_out_ of the VO_*x*_-based circuit with *R*_L_ of 15 kΩ under different parameters of *V*_in_. The corresponding *V*_out_ under these conditions is presented in Figure S13a–c (in the Supporting Information), respectively. Equivalent measurements using *R*_L_ values of 10 kΩ and 22 kΩ are shown in Figures S14 and S15 (in the Supporting Information), respectively. In addition, the TTFS extracted from Figure 5g–i is summarized in Figure 5j–l, respectively.

The results demonstrate that the LIF response can be effectively tuned by adjusting the input pulse parameters. Increasing either the pulse amplitude or pulse width accelerates charging of *C*_parasitic_, resulting in a shorter TTFS with faster neuronal response. Conversely, extending the pulse interval leads to a longer TTFS due to increased charge dissipation through the *C*_parasitic_ of the VO_*x*_ TSM. Additionally, a larger *R*_L_ reduces the input current, which slows down the charging process of *C*_parasitic_ and consequently delays or even suppresses the firing response. Within the framework of neuromorphic computing, *R*_L_ serves as an analogue of synaptic weight. Under identical stimulation conditions, a larger *R*_L_ corresponding to a smaller synaptic weight decreases the rate of membrane potential accumulation, leading to a longer TTFS.

Beyond temporal modulation, the circuit also exhibits spatial and spatiotemporal processing capability, as demonstrated in Figure S16 (in the Supporting Information). To achieve this, the neuronal circuit is modified by introducing two input resistors (*R*_L_1 and *R*_L_2) connected to the VO_*x*_ TSM device, thereby forming two independent input terminals, as shown in Figure S16a in the Supporting Information. Voltage pulses with an amplitude of 3.25 V, a width of 500 ns, and an interval of 300 ns are applied to these terminals. When a pulse train is delivered to only one input terminal (through either *R*_L_1 and *R*_L_2), no output current spikes are observed at the ground terminal, confirming that a single input is insufficient to trigger firing (Figure S16b,c in the Supporting Information). In contrast, when both inputs receive pulses simultaneously, the neuron exhibits active spiking, demonstrating that the device integrates signals from the two independent inputs and achieves spatial summation (Figure S16d in the Supporting Information).

To further validate spatiotemporal summation, an experiment is designed with staggered inputs. As shown in Figure S16e in the Supporting Information, the pulse train *V*_in_2 is applied 8.1 μs earlier than *V*_in_1. Active neuronal firing is observed only during the overlapping period of the two pulse trains (approximately 8–16 μs). This result confirms that the VO_*x*_-based LIF neuron model integrates signals arriving from different inputs at different times, thereby reproducing the spatiotemporal summation behavior characteristic of biological neurons. This behavior also demonstrates that the circuit successfully reproduces the strength-modulated spike frequency response observed in biological neurons.

In order to further assess its suitability for human-machine interface applications, the energy efficiency of the circuit is evaluated by estimating the energy consumption per spike. A minimum energy of approximately 4.7 nJ per spike is achieved, as summarized in Table S3 (in the Supporting Information). These findings highlight the circuit’s capability to process analog signals in a highly tunable and efficient manner, supporting its applicability in advanced neurorobotic systems.^53^

## 3 Conclusions

In summary, we have demonstrated VO_*x*_ TSM that intrinsically supports dual neuronal modes, functioning as both a spiking encoder and a leaky integrate-and-fire (LIF) unit. The developed device exhibits nonlinear switching behavior governed by a thermally driven IMT, with temperature-dependent electrical and XRD measurements confirming a strong correlation between structural phase evolution and electrical response. When integrated into a passive circuit, the VO_*x*_ TSM enables dual-mode operation, delivering high spiking frequency, rapid response, and low energy consumption. This functionality is achieved without relying on complex peripheral circuitry, highlighting its suitability for highly scalable neuromorphic architectures. The demonstrated performance underscores the close interplay between phase transition dynamics and neuromorphic behavior, providing a compact and energy-efficient pathway for implementing biologically inspired computation. These findings pave the way for multifunctional neuron devices and offer a promising platform for future spiking neural network hardware.

## 4 Experimental Section

### 4.1 Device Fabrication

A 137 nm-thick VO_*x*_ film was deposited on a commercial Si substrate precoated with Ti (10 nm)/Pt (100 nm) layers (CrysTec GmbH) using RF magnetron sputtering. Prior to deposition, the sputtering chamber was evacuated to a base pressure of approximately 5 × 10^–7^ mbar to minimize residual gas contamination and ensure high-purity, defect-reduced film growth with good reproducibility. The deposition was carried out using a high-purity (99.99%) V_2_O_5_ ceramic target under an argon atmosphere with a flow rate of 3 sccm, maintaining a working pressure of 5 × 10^–3^ mbar. The substrate was held at 450 °C during the process to promote proper crystallinity and stoichiometry in the resulting VO_*x*_ film. After deposition, the sample was allowed to cool to room temperature under the same ambient conditions. A lift-off process based on photolithography was used to define the top electrode area of 30 × 30 μm^2^. Subsequently, a 70 nm-thick Pt top electrode was sputtered onto the patterned sample, followed by the deposition of a 200 nm-thick Au capping layer using a Quorum sputter coater. Finally, the lift-off step was performed using acetone to remove the excess metal, resulting in well-defined Au/Pt electrodes on the VO_*x*_ surface.

### 4.2 Material and Electrical Characterization

Cross-sectional transmission electron microscopy (TEM), electron energy loss spectroscopy (EELS), and energy-dispersive X-ray spectroscopy (EDS) were performed using a [EM000800] JEOL JEM-2100F Cs STEM. X-ray diffraction (XRD) analysis was conducted using Cu K_α_ radiation on a Rigaku SmartLab diffractometer operated in parallel beam geometry. X-ray photoelectron spectroscopy (XPS) measurements were carried out in the DAISY-BAT laboratory,^45^ using a PHI 5000 VersaProbe multifunctional photoelectron spectroscopy system (Physical Electronics (PHI)), which is equipped with a semispherical spectrometer, monochromatic Al Kα X-ray source (*h*ν = 1486.7 eV), and a dual beam charge compensation system consisting of a low energy ion gun and an electron flood. Electrical measurements were conducted in ambient conditions at room temperature using an Agilent B1530A waveform generator/fast measurement unit (WGFMU) and a Keysight B1500A semiconductor parameter analyzer. Any current flowing into the instrument’s ground terminal is defined as a negative value. Output voltage waveforms of the VO_*x*_ TSM devices were recorded using a KEYSIGHT MXR204B Mixed Signal Oscilloscope. The observed superficial signal noise observed in the LIF data is primarily attributed to signal interference between the Keysight B1500A analyzer and the KEYSIGHT MXR204B oscilloscope, resulting from the measurement configuration. Importantly, this interference does not substantially affect the underlying LIF behavior of the device.

## Supplementary Material



## Notes

V 2p and O 1s photoelectron spectra of the V_2_O_5_ and VO_*x*_ thin-films; HAADF image of the Pt/VO_*x*_/Pt TSM device with corresponding EDS mapping and line-scan analysis; temperature-dependent resistivity of the VO_*x*_ thin film during heating and cooling cycles; *I*–*V* characteristics of 15 individual VO_*x*_ TSM devices; extracted positive and negative threshold voltages (*V*_th_pos_, *V*_th_neg_) and holding voltages (*V*_h_pos_, *V*_h_neg_) for 15 individual VO_*x*_ TSM devices; comparison of the pristine and after-heating-cycle *I*–*V* characteristics of the VO_*x*_ TSM device; switching speed characteristics of the VO_*x*_ TSM device under a 1.5 V voltage pulse; output oscillation responses of *V*_out_ and *I*_out_ under negative triangular *V*_in_ with varying *R*_L_; corresponding *V*_out_ responses under 100 μs rectangular *V*_in_ with different *R*_L_ values; output oscillation responses of *V*_out_, *I*_out_, and spiking frequency under sinusoidal *V*_in_; *C*–*V* measurement of Pt/VO_*x*_/Pt TSM parasitic capacitance; corresponding *V*_out_ responses under variations in *V*_in_, *t*_d_, and Δ*t*, with the pulse number fixed at 30 and *R*_L_ = 15 and 22 kΩ; spatial and spatiotemporal summation by utilizing the VO_*x*_-based LIF neuron model; tables for the binding energies of vanadium 2p_3/2_ valence states; and the summary of C2C and D2D variations in the electrical characteristics of VO_*x*_ TSM devices (PDF)
