# Supplementary material for: Thermally Activated Negative Differential Resistance VO x Memristor with Switchable Rate and Leaky Integrate-and-Fire Spiking Dynamics
Source: ACS Nano. 2025 Oct 13;19(42):37165–74. doi: 10.1021/acsnano.5c11481 (PMC12574203; doi:10.1021/acsnano.5c11481)
Supplement: Supplementary file 1 [file nn5c11481_si_001.pdf]

Supporting Information

Thermally Activated Negative Differential Resistance VO_x Memristor with Switchable Rate and Leaky Integrate-and-Fire Spiking Dynamics

Li-Chung Shih[a], Zih-Siao Liao[a], Gennady Cherkashinin[b], Eszter Piros[b], Lambert Alff[b,*], and Jen-Sue Chen[a,c,*]

[a] Department of Materials Science and Engineering, National Cheng Kung University, Tainan 70101, Taiwan

[b] Advanced Thin Film Technology Division, Institute of Materials Science, Technische Universität Darmstadt, Peter-Grünberg-Str. 2, Darmstadt 64287, Germany

[c] Program on Semiconductor Packaging and Testing, Academy of Innovative Semiconductor and Sustainable Manufacturing, National Cheng Kung University, Tainan 70101, Taiwan

[*] Corresponding author:
Lambert Alff: lambert.alff@tu-darmstadt.de
Jen-Sue Chen: jenschen@ncku.edu.tw

KEYWORDS: VO_x threshold switching neuron memristor, insulator-to-metal transition, negative differential resistance, spiking encoder, and leaky integrate-and-fire neuron model

Supplementary Note 1:

The XPS measurements were performed at the DAISY-BAT laboratory[1] using a PHI 5000 VersaProbe system (Physical Electronics, PHI). The system is equipped with a monochromatic Al Kα X-ray source (hν=1486.7 eV), a hemispherical energy analyzer, and a dual-beam charge compensation system consisting of a low-energy ion gun and electron flood source. The X-ray spot size was 200 μm, and measurements were performed with a pass energy of 23.5 eV and an electron take-off angle of 45°. The base pressure during acquisition was maintained below 10^{-9} mbar. All binding energies were calibrated with respect to the Fermi level of a sputtered Ag or Au reference. Background subtraction was performed using a Shirley-type function.

Quantitative elemental analysis was carried out using the following expression:

$$C_A = \frac{n_A}{\sum n_i} = \frac{I_A/ASF_A}{\sum I_i/ASF_i} \times 100\%$$

where C_A is the relative concentration of element A, n_A is the atomic concentration of element A, ASF_A is the atomic sensitivity factor of element A and I_A is the intensity of the characteristic photoelectron line of the element A. By using the atomic sensitivity factor, an error in the quantitative analysis can be around 15%. As an example, the in-situ measured V 2p spectrum of a V_2O_5 thin film is shown in Figure SN1 (in the Supporting Information). The sharp V $2p_{3/2}$ peak confirms the V^{5+} oxidation state, and the calculated stoichiometry from the XPS data is approximately 2.4. Given the known uncertainty in XPS quantification, this value is in good agreement with the expected $VO_{2.2}$ composition, confirming that the in-situ XPS measurement accurately reflects the bulk stoichiometry of the deposited film.

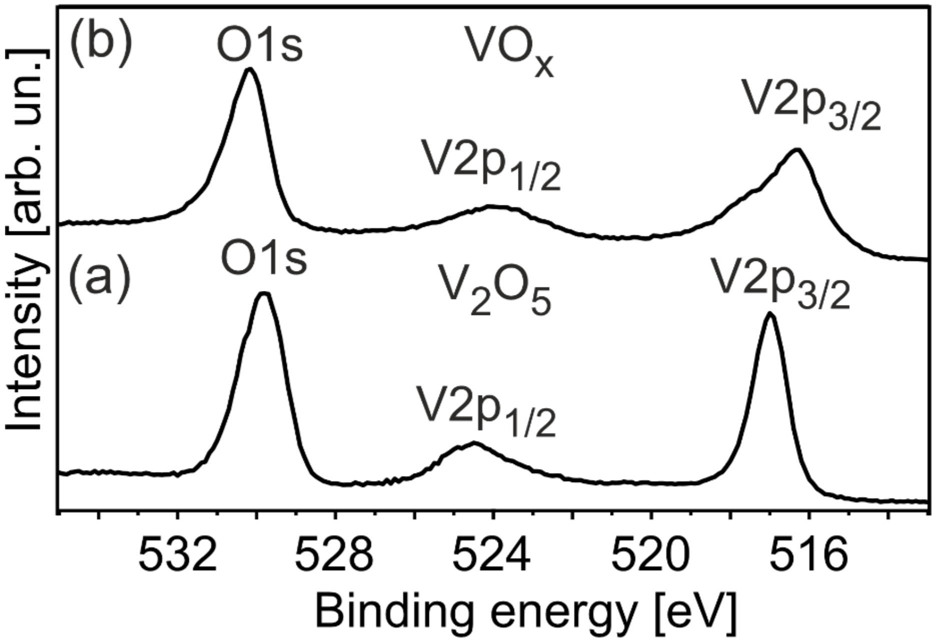

Figure SN1. The V 2p and O 1s X-ray photoelectron spectra of (a) V_2O_5 and (b) $VO_{2.2}$ thin-films. The spectral characteristics are in a good agreement with earlier published work on the electronic structure of various vanadium oxides.[2]

Supplementary Note 2: Role of R_L and its relationship to device resistance

The LIF behavior is governed by the interaction between the parasitic capacitance (Cparasitic) and the threshold switching of the memristor. During each input pulse, Cparasitic charges and incrementally raises the voltage across the memristor, while leakage during the pulse intervals gradually reduces it. When the accumulated voltage across the memristor exceeds Vth, the memristor switches to the on-state, leading to a rapid discharge of Cparasitic and generating a current spike. The voltage then drops below Vh, resetting the memristor to its off-state and enabling a new integration cycle.

The relation between R_L and the device resistance can be described by simple voltage division:

$$\begin{cases} \textbf{Charging}: V_{device} = V_{input} \times \dfrac{R_{HRS}}{R_L + R_{HRS}} > V_{th} & (1) \\ \textbf{Discharging}: V_{device} = V_{input} \times \dfrac{R_{LRS}}{R_L + R_{LRS}} < V_h & (2) \end{cases}$$

Equation (1) ensures that the voltage across the device exceeds Vth to trigger threshold switching and turn on the device, while the equation (2) ensures that the voltage could drop below Vhold for the device to reset and re-integrate. These equations indicate that the proper choice of R_L is inherently constrained by the device resistances in both HRS and LRS.

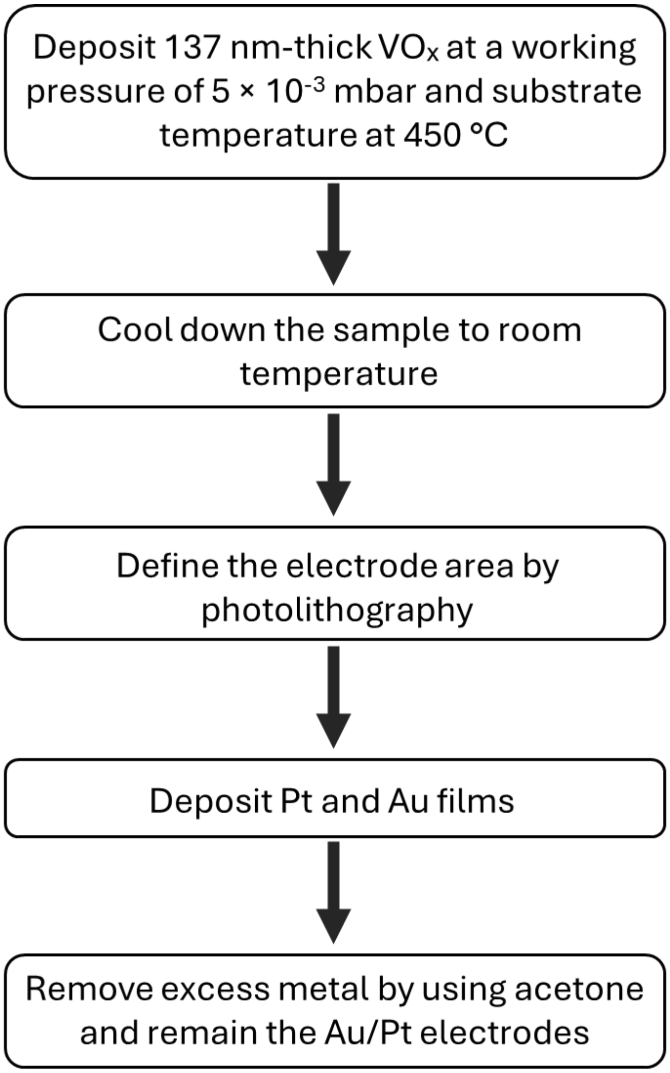

Figure S1. The process flow chart of the Pt/VO_x/Pt TSM device.

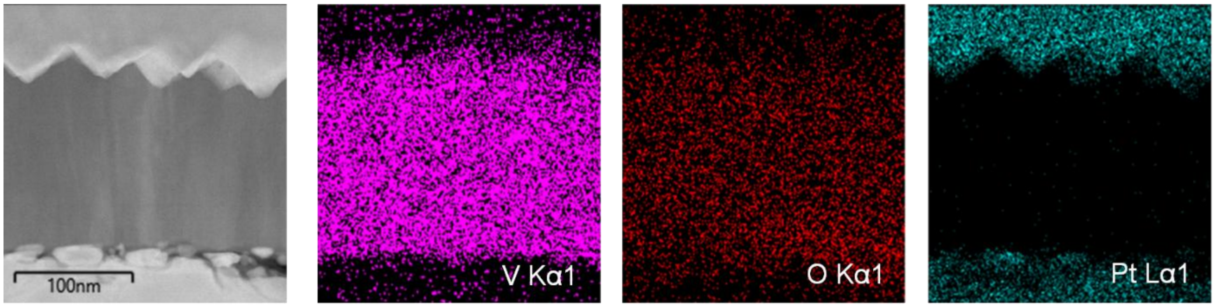

Figure S2. HAADF image of the $Pt/VO_x/Pt$ TSM device with corresponding EDS mapping analysis of Pt, V, and O elements.

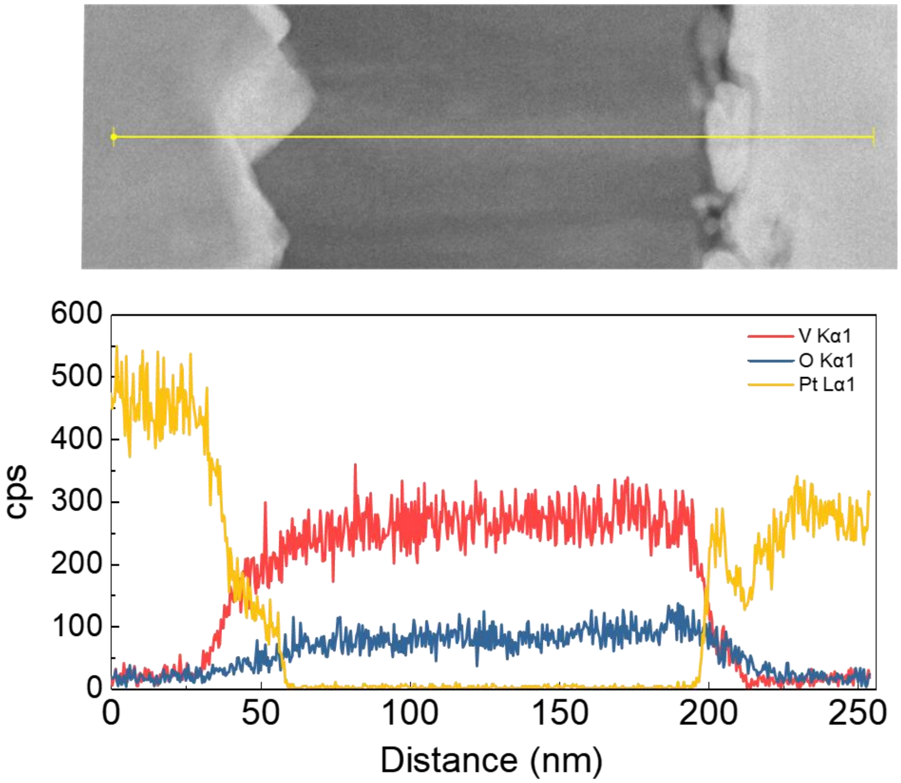

Figure S3. HAADF image of the Pt/VO_x/Pt TSM device with corresponding EDS line scan analysis of Pt, V, and O elements.

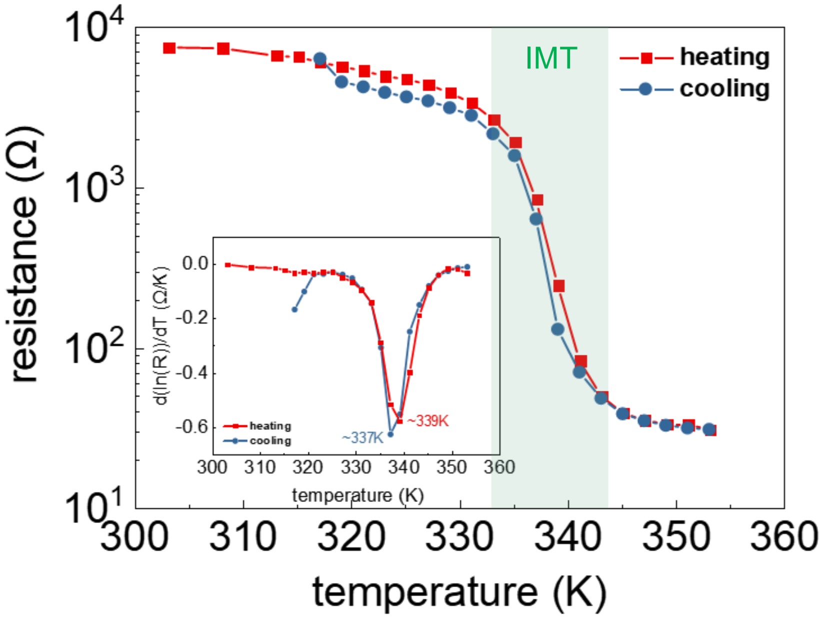

Figure S4. Temperature-dependent resistivity of the VO_x thin film during heating and cooling cycles. Inset: Gaussian-fitted derivative plots of d(log R)/dT versus temperature, revealing the transition temperatures for both heating (~339 K) and cooling (~337 K) processes.

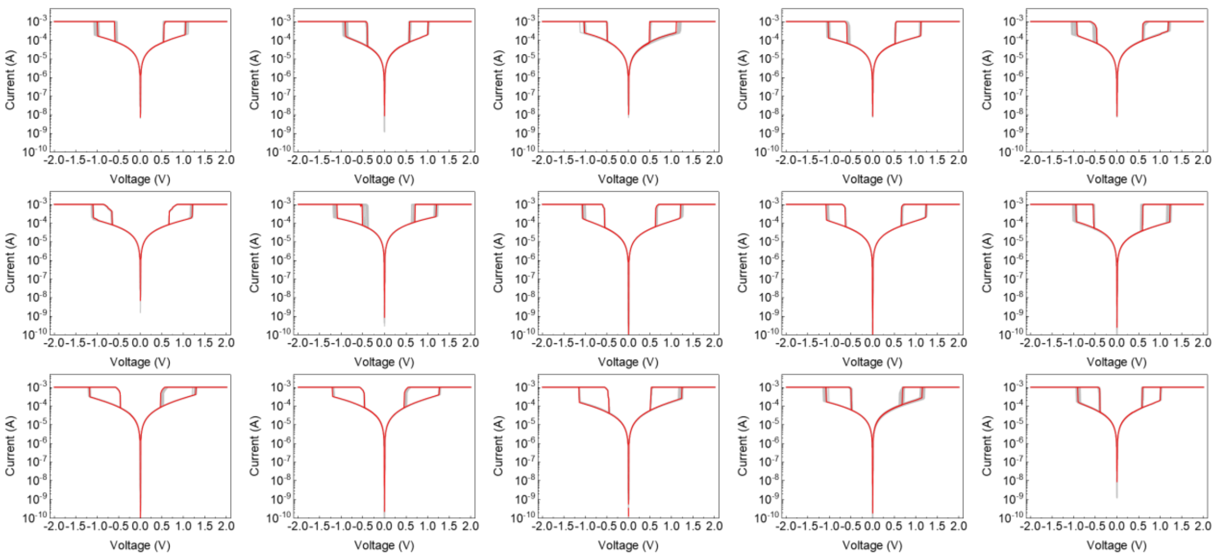

Figure S5. I-V characteristics of 15 individual VO_x TSM devices, each measured over 20 consecutive switching cycles.

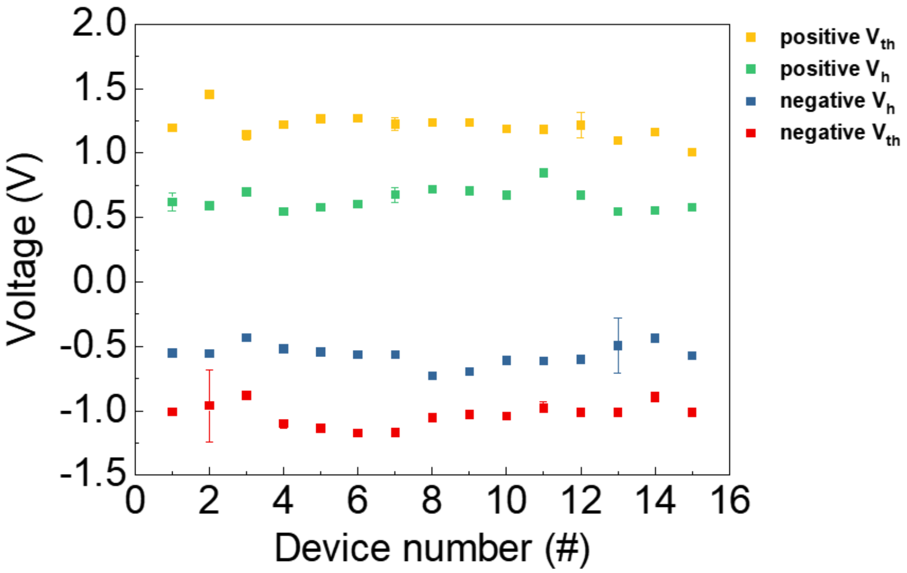

Figure S6. Extracted positive and negative threshold voltages (V_{th_pos}, V_{th_neg}) and holding voltages (V_{h_pos}, V_{h_neg}) for 15 individual VO_x TSM devices. Error bars represent standard deviations over 20 repeated switching cycles.

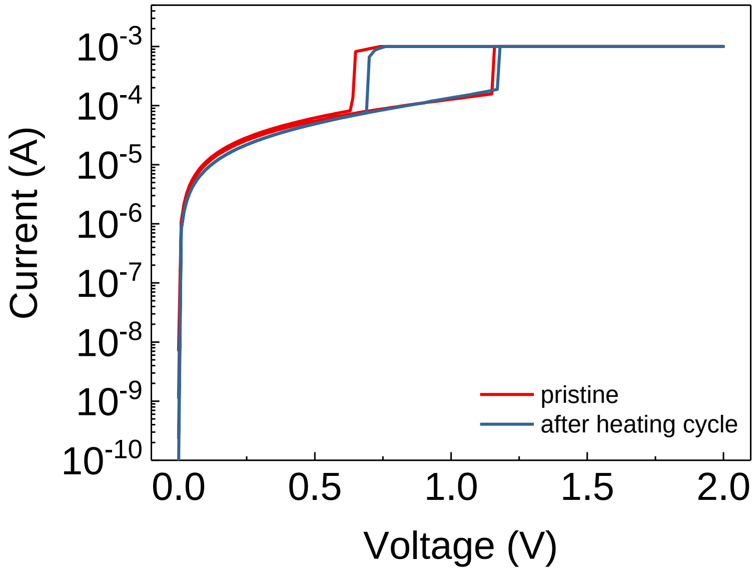

Figure S7. Comparison of the pristine and after-heating-cycle I–V characteristics of the VO_x TSM device, demonstrating reversible and stable electrical switching behavior.

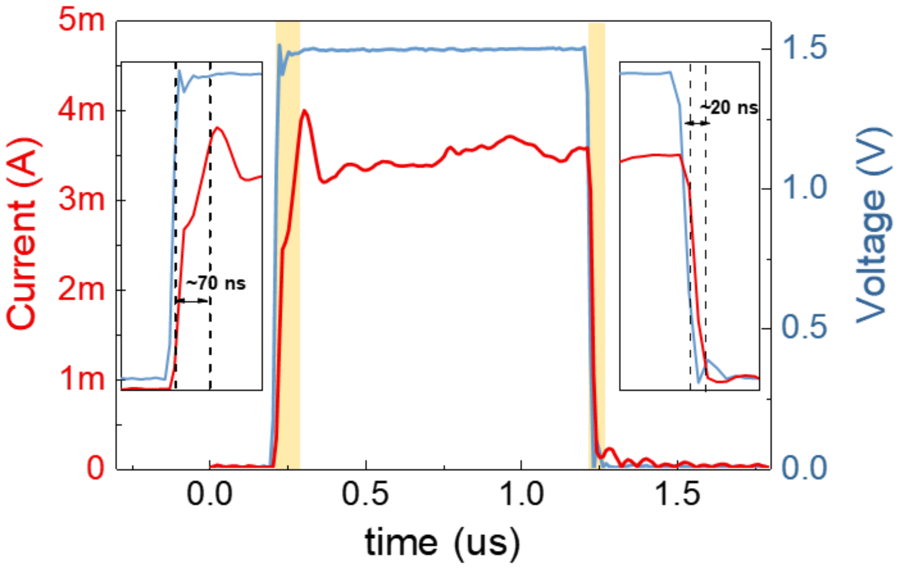

Figure S8. Switching speed characteristics of the VO_x TSM device under a 1.5 V voltage pulse.

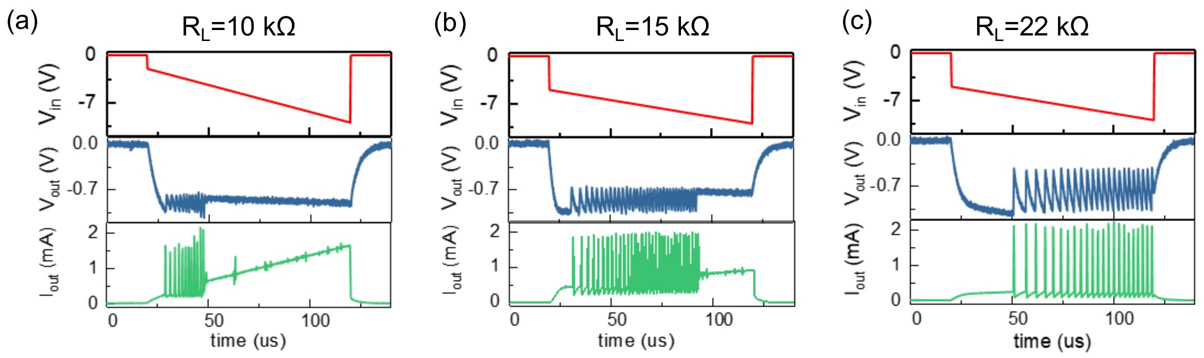

Figure S9. Output oscillation responses of V_{out} and I_{out} under negative triangular V_{in} with varying R_L.

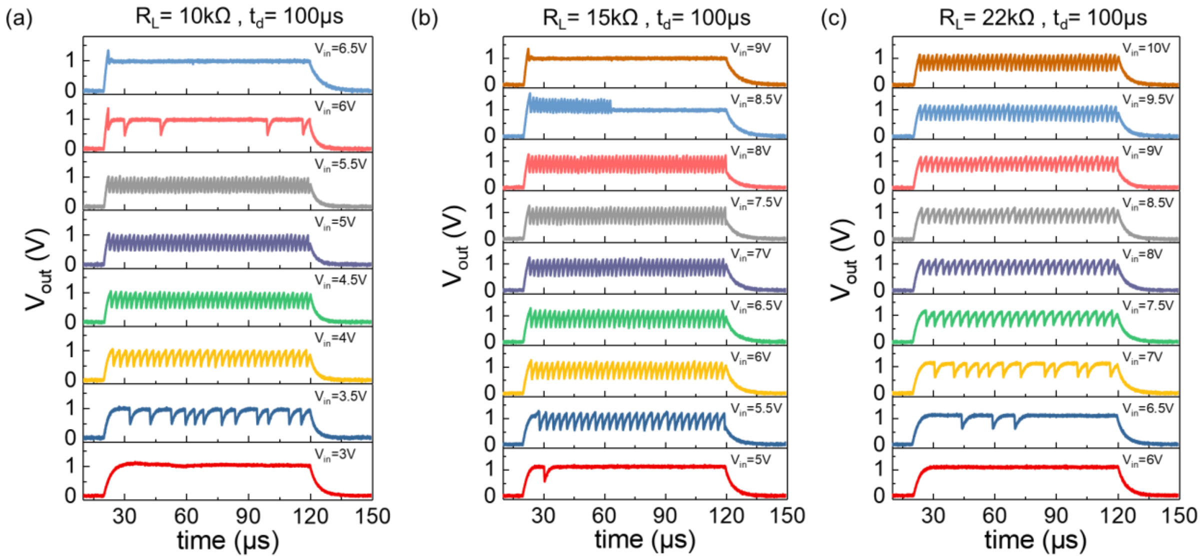

Figure S10. Corresponding V_{out} responses under 100 μs rectangular V_{in} with different R_L values.

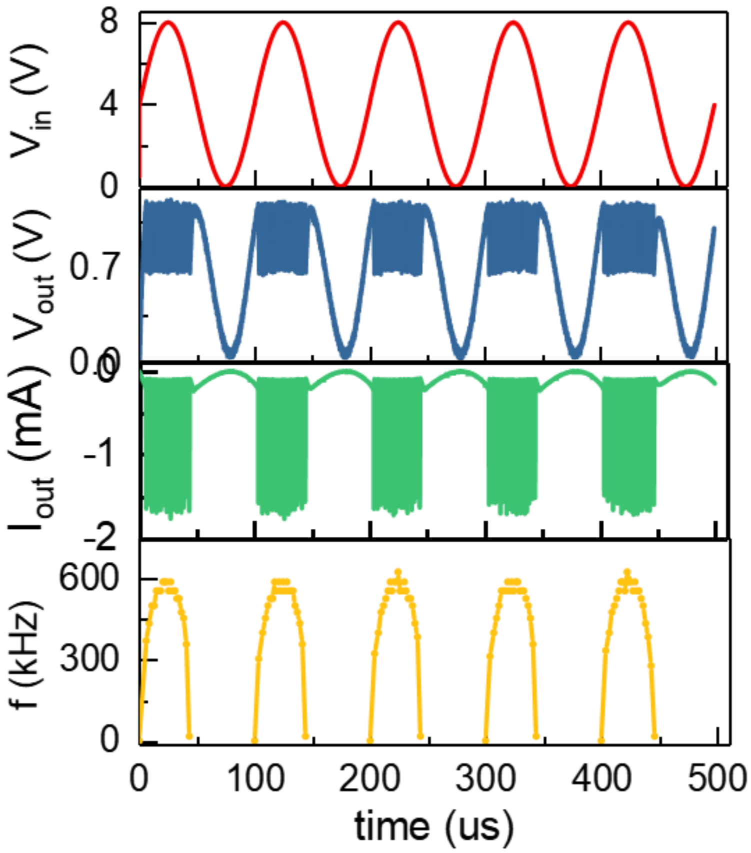

Figure S11. Output oscillation responses of V_{out}, I_{out}, and spiking frequency under sinusoidal V_{in} with R_L of 15 kΩ.

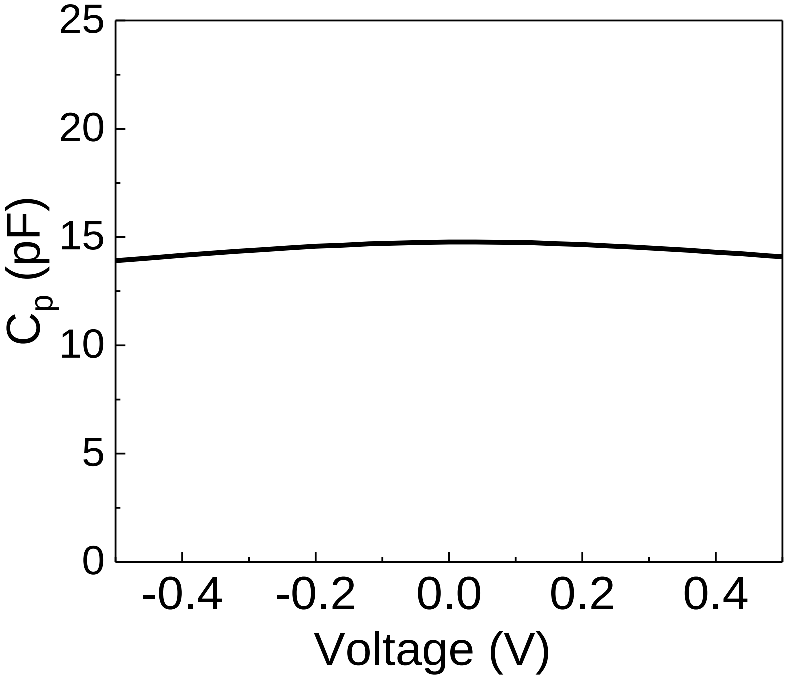

Figure S12. The C-V measurement of Pt/VO_x/Pt TSM parasitic capacitance, where the AC frequency is 500 kHz and the oscillating level is 100 mV. The measured parasitic capacitance of VO_x memristor is 14 pF.

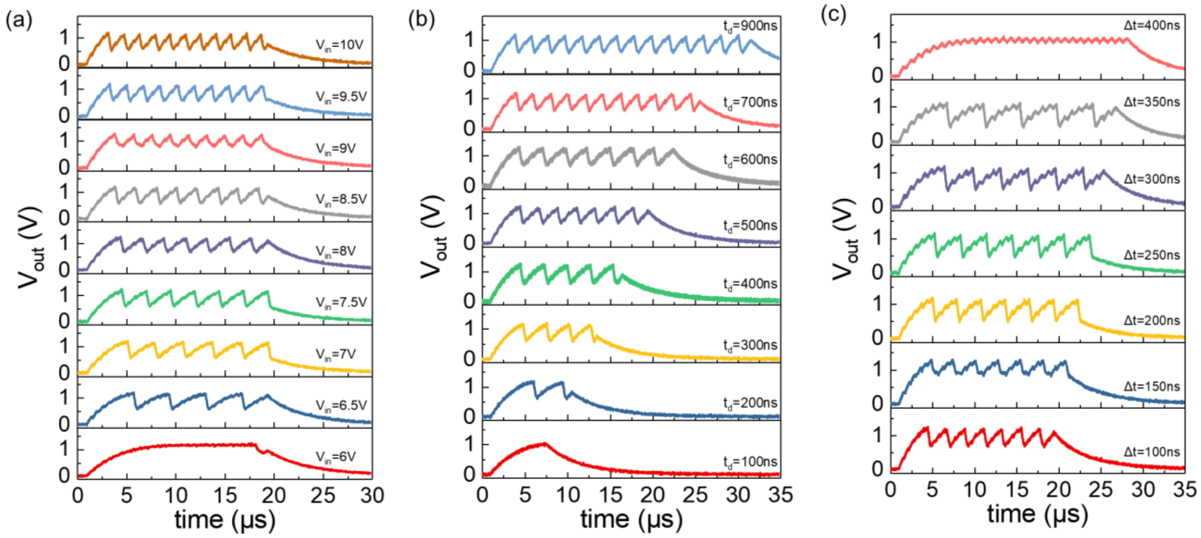

Figure S13. Corresponding V_{out} responses under variations in (a) V_{in}, (b) t_d, and (c) Δt, with the pulse number fixed at 30 and R_L = 15 kΩ.

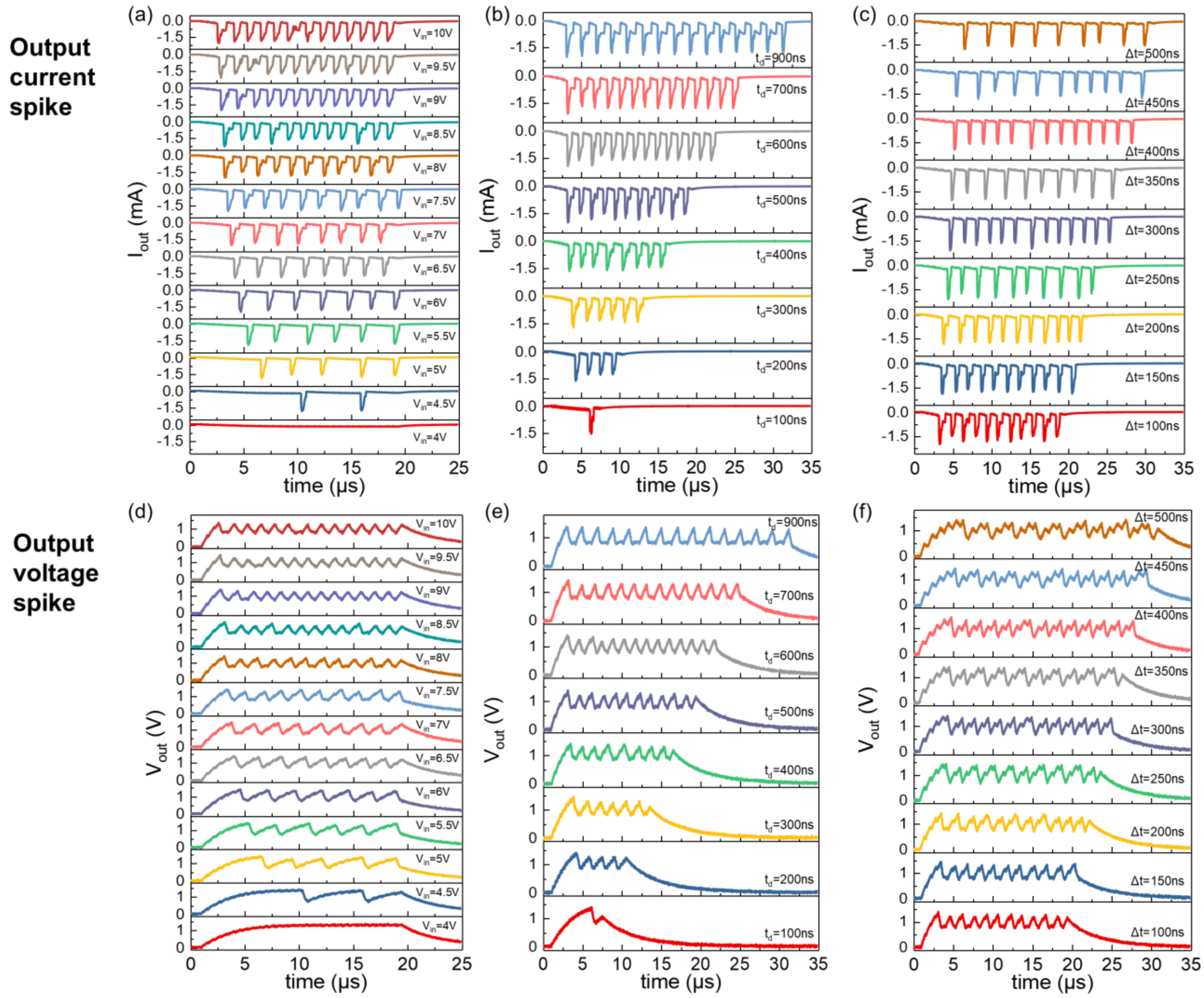

Figure S14. Corresponding I_{out} and V_{out} responses under variations in (a, d) V_{in}, (b, e) t_d, and (c, f) Δt, with the pulse number fixed at 30 and R_L = 10 kΩ.

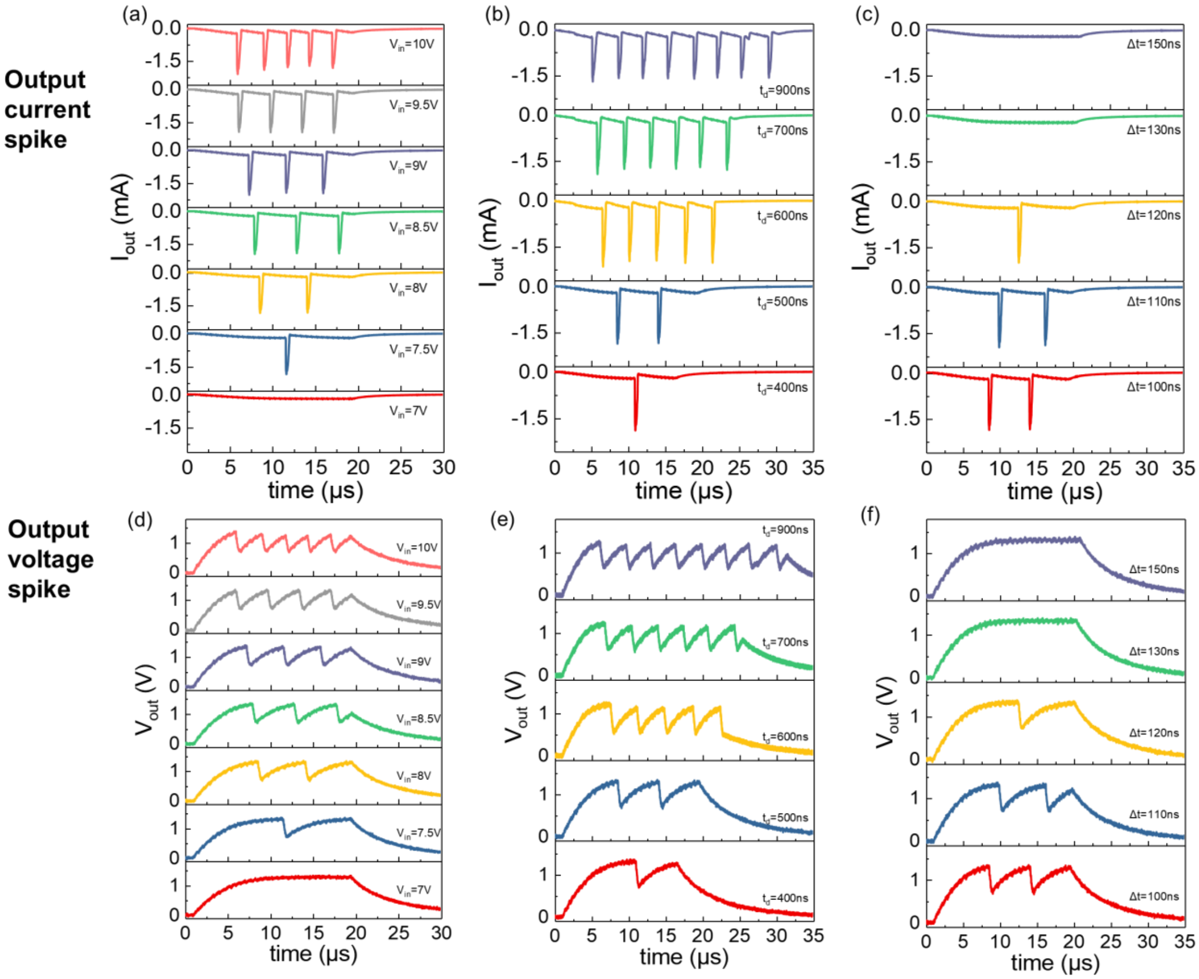

Figure S15. Corresponding I_{out} and V_{out} responses under variations in (a, d) V_{in}, (b, e) t_d, and (c, f) Δt, with the pulse number fixed at 30 and R_L = 22 kΩ.

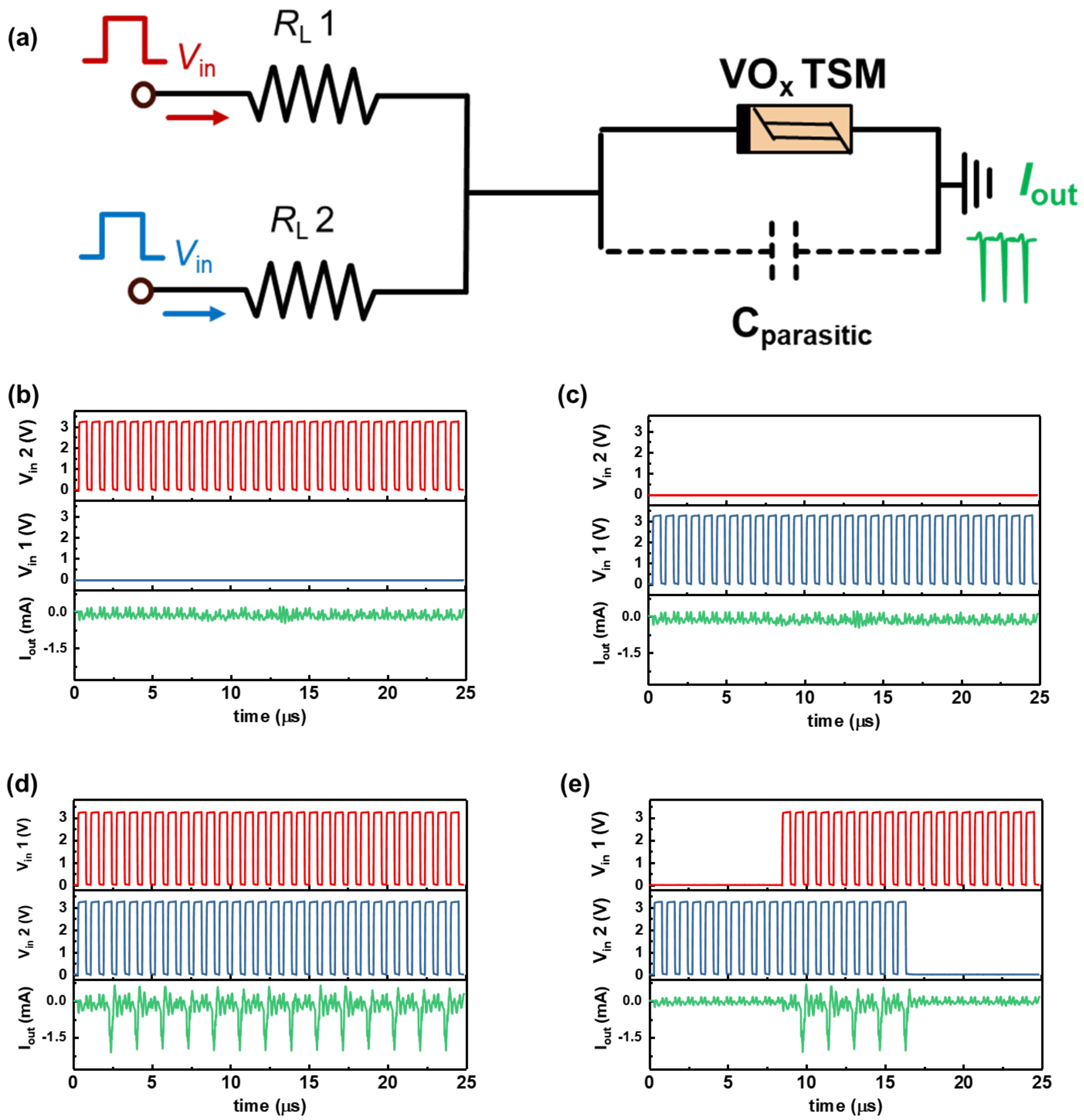

Figure S16. Spatial and spatiotemporal summation by utilizing the VO_x-based LIF neuron model. (a) Schematic of the two-input neuron circuit. A single input via (b) R_L1 and (c) R_L2 is insufficient to trigger firing. (d) Simultaneous inputs on both R_L1 and R_L2 result in firing, demonstrating spatial summation. (e) The neuron fires only during the overlapping period, demonstrating spatiotemporal summation.

Table S1. Binding energies of vanadium $2p_{3/2}$ valence states corresponding to different vanadium oxidation states in the as-grown VO_x film deposited on a Si substrate.

	V^{3+}	V^{4+}	V^{5+}
Binding energy (eV)	515.5	516.6	518.0
FWHM	1.41	1.41	1.41
Area	2522.94	12460.4	9946.9
Concentration (at%)	12.5	51.1	36.4

Table S2. Summary of C2C and D2D variations in the electrical characteristics of VO_x TSM devices.

	C2C variation				D2D variation			
Electrical characteristics	V_{th_pos}	V_{h_pos}	V_{th_neg}	V_{h_neg}	V_{th_pos}	V_{h_pos}	V_{th_neg}	V_{h_neg}
Mean value (μ)	1.20 V	0.62 V	-1.00 V	-0.55 V	1.20V	0.64V	-1.03V	-0.56V
Standard deviation (σ)	0.02 V	0.07 V	0.02 V	0.03 V	0.09 V	0.0 8V	0.08 V	0.07 V
Coefficient of variation	2.2%	11.5%	2.6%	5.9%	7.7%	12.5%	8.2%	13.7%

Table S3. Comparison of the artificial neuron memristor for the implementation of encoder and LIF models.

Device structure	V_{th}/V_h (V)	Energy consumption	Switching speed on/off	Highest spike frequency (encoder)	Shortest TTFS (LIF model)	Ref.
Pt/VO_x/Pt	1.2/0.6 V	4.7 nJ/spike	70/20 ns	570 kHz	1.6 us	This work
Au/VO_2/Au	1.35/0.8 V	2.9 nJ/spike	200/75 ns	1.3 MHz	x	[3]
Au/VO_2/Au	3.4/1.5V	6.92 nJ/spike	70 /60 ns	~480 kHz	x	[4]
Au/VO_2/Au	1.7/0.75V	3.9 nJ/spike	x	650 kHz	x	[5]
Pt/V_3O_5/Pt	1.13/0.93 V	~190 nJ/spike	x	82 kHz	x	[6]
Au/VO_2/Au	3.2/1.5 V	x	x	x	~50 us	[7]
Pt/VO_2/Al_2O_3/Pt	2.5/1.4 V	x	x	174 kHz	x	[8]
Pt/VO_2/Pt	0.6/0.2 V	3 nJ/spike	x	16.8 kHz	x	[9]
Pt/NbO_x/Pt	2.3/1.6V	0.9 nJ/spike	~60 /100 ns	8 MHz	x	[10]
TiN/NbO_x/Pt	2.0/0.23 V	x	44/~35 ns	x	32 ms	[11]
Pt/NbO_x/Pt	0.8/0.6V	x	50/25 ns	x	2.1 us	[12]
Ag/TaO_x/ITO	~0.3/0.1V	0.5 uW/spike	10/40 ns	x	~170 ms	[13]

References

(1) Hausbrand, R.; Cherkashinin, G.; Ehrenberg, H.; Gröting, M.; Albe, K.; Hess, C.; Jaegermann, W. Fundamental degradation mechanisms of layered oxide Li-ion battery cathode materials: Methodology, insights and novel approaches. *Materials Science and Engineering: B* **2015**, *192*, 3-25, DOI: 10.1016/j.mseb.2014.11.014.

(2) Wu, Q.-H.; Thissen, A.; Jaegermann, W.; Liu, M. Photoelectron spectroscopy study of oxygen vacancy on vanadium oxides surface. *Appl. Surf. Sci.* **2004**, *236*(1), 473-478, DOI: 10.1016/j.apsusc.2004.05.112.

(3) Yuan, R.; Duan, Q. X.; Tiw, P. J.; Li, G.; Xiao, Z. J.; Jing, Z. K.; Yang, K.; Liu, C.; Ge, C.; Huang, R.; Yang, Y. C. A calibratable sensory neuron based on epitaxial VO_2 for spike-based neuromorphic multisensory system. *Nat. Commun.* **2022**, *13*(1), 3973, DOI: 10.1038/s41467-022-31747-w.

(4) Yuan, R.; Tiw, P. J.; Cai, L.; Yang, Z. Y.; Liu, C.; Zhang, T.; Ge, C.; Huang, R.; Yang, Y. C. A neuromorphic physiological signal processing system based on VO_2 memristor for next-generation human-machine interface. *Nat. Commun.* **2023**, *14*(1), 3695, DOI: 10.1038/s41467-023-39430-4.

(5) Li, Z. Y.; Li, Z. S.; Tang, W.; Yao, J. P.; Dou, Z. P.; Gong, J. J.; Li, Y. F.; Zhang, B. N.; Dong, Y. X.; Xia, J.; Sun, L.; Jiang, P.; Cao, X.; Yang, R.; Miao, X. S.; Yang, R. G. Crossmodal sensory neurons based on high-performance flexible memristors for human-machine in-sensor computing system. *Nat. Commun.* **2024**, *15*(1), 7275, DOI: 10.1038/s41467-024-51609-x.

(6) Nath, S. K.; Das, S. K.; Nandi, S. K.; Xi, C.; Marquez, C. V.; Rúa, A.; Uenuma, M.; Wang, Z. R.; Zhang, S. Q.; Zhu, R. J.; Eshraghian, J.; Sun, X.; Lu, T.; Bian, Y.; Syed, N.; Pan, W. W.; Wang, H.; Lei, W.; Fu, L.; Faraone, L.; Liu, Y.; Elliman, R. G. Optically Tunable Electrical Oscillations

in Oxide-Based Memristors for Neuromorphic Computing. *Adv. Mater.* **2024**, *36*(25), 2400904, DOI: 10.1002/adma.202400904.

(7) Deng, S. B.; Yu, H. M.; Park, T. J.; Islam, A. N. M. N.; Manna, S.; Pofelski, A.; Wang, Q.; Zhu, Y. M.; Sankaranarayanan, S. K. R. S.; Sengupta, A.; Ramanathan, S. Selective area doping for Mott neuromorphic electronics. *Sci. Adv.* **2023**, *9*(11), eade4838, DOI: 10.1126/sciadv.ade4838.

(8) Fang, S. L.; Han, C. Y.; Han, Z. R.; Ma, B.; Cui, Y. L.; Liu, W.; Fan, S. Q.; Li, X.; Wang, X. L.; Zhang, G. H.; Huang, X. D.; Geng, L. An Artificial Spiking Afferent Neuron System Achieved by 1M1S for Neuromorphic Computing. *IEEE Trans. Electron Devices* **2022**, *69*(5), 2346-2352, DOI: 10.1109/TED.2022.3159270.

(9) Han, C. A. Y.; Han, Z. R.; Fang, S. L.; Fan, S. Q.; Yin, J. Q.; Liu, W. H.; Li, X.; Yang, S. Q.; Zhang, G. H.; Wang, X. L.; Geng, L. Characterization and Modelling of Flexible VO_2 Mott Memristor for the Artificial Spiking Warm Receptor. *Adv. Mater. Interfaces* **2022**, *9*(19), 2200394, DOI: 10.1002/admi.202200394.

(10) Zhong, S.; Zhang, Y. S.; Zheng, H.; Yu, F. W.; Zhao, R. Spike-Based Spatiotemporal Processing Enabled by Oscillation Neuron for Energy-Efficient Artificial Sensory Systems. *Adv. Intell. Syst.* **2022**, *4*(9), 2200076, DOI: 10.1002/aisy.202200076.

(11) Zhao, J. H.; Tong, L.; Niu, J. Z.; Fang, Z. L.; Pei, Y. F.; Zhou, Z. Y.; Sun, Y.; Wang, Z. R.; Wang, H.; Lou, J. Z.; Yan, X. B. A bidirectional thermal sensory leaky integrate-and-fire (LIF) neuron model based on bipolar NbO_x volatile threshold devices with ultra-low operating current. *Nanoscale* **2023**, *15*(43), 17599-17608, DOI: 10.1039/d3nr03034b.

(12) Duan, Q. X.; Jing, Z. K.; Zou, X. L.; Wang, Y. H.; Yang, K.; Zhang, T.; Wu, S.; Huang, R.; Yang, Y. C. Spiking neurons with spatiotemporal dynamics and gain modulation for

monolithically integrated memristive neural networks. *Nat. Commun.* **2020**, *11*(1), 3399, DOI: 10.1038/s41467-020-17215-3.

(13) Chen, C. S.; He, Y. L.; Mao, H. W.; Zhu, L.; Wang, X. J.; Zhu, Y.; Zhu, Y. X.; Shi, Y.; Wan, C. J.; Wan, Q. A Photoelectric Spiking Neuron for Visual Depth Perception. *Adv. Mater*. **2022**, *34*(20), 2201895, DOI: 10.1002/adma.202201895.
